# Linking new information to a short-lasting memory trace induces consolidation in the hippocampus

**DOI:** 10.1016/j.isci.2024.111320

**Published:** 2024-11-05

**Authors:** Andressa Gabriela Soliani, Jessica Santos Baptista, Beatriz Gangale Muratori, Lucia Armelin Correa, Suzete Maria Cerutti

**Affiliations:** 1 Cellular and Behavioral Pharmacology Laboratory, Department of Biological Sciences, Federal University of Sao Paulo, Diadema, São Paulo, Brazil; 2 Department of Biological Sciences, Federal University of Sao Paulo, Diadema, São Paulo, Brazil

**Keywords:** Cognitive neuroscience, Molecular neuroscience, Neuroscience

## Abstract

Novelty often influences the retention of nearby weak and transient memory traces, yet its precise role in shaping long-term memory storage remains elusive. Here, we demonstrate that a short-lasting memory is stabilized into a long-lasting one when new information is linked to the weak mnemonic trace in rats, resulting in the formation of long-term memories that are recalled together. An increased overlap between neuronal ensembles and *de novo* protein synthesis in the dorsal CA1 region of the hippocampus (dCA1) mediates this process. This intricate interconnectedness relies on both temporal and contextual relations between experiences, enhancing the adaptive value of memory consolidation. Finally, this phenomenon is negatively affected by aging, which is associated with reduced ensemble size after novelty exposure and diminished overlap between ensembles in aged dCA1. These findings provide valuable insights into the selectivity and malleability of memory consolidation and its decline during aging.

## Highlights

• New information is linked to a short-lasting memory, inducing memory consolidation
• This process involves increased ensemble overlap and protein synthesis in the dCA1 region
• Aging reduces ensemble overlap and size in the dCA1, resulting in consolidation failure

## Graphical abstract

**Figure:**
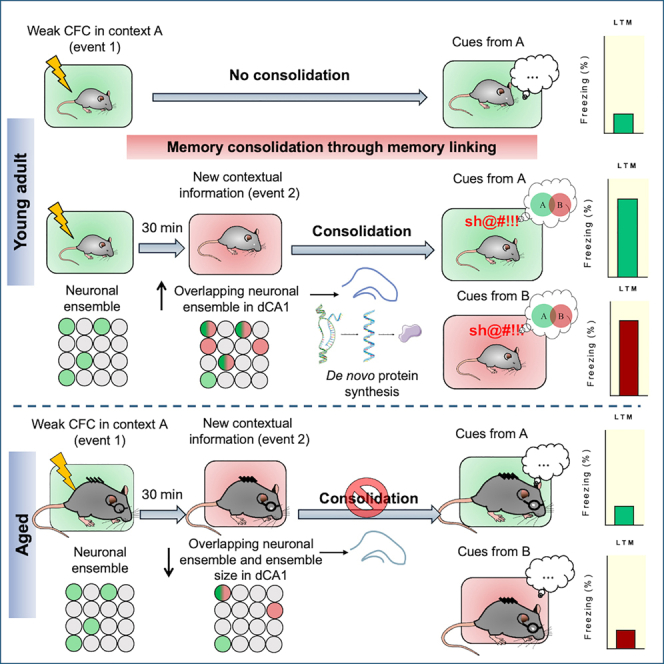

## Introduction

The ability to encode and store information for later recall is fundamental to our existence and identity. However, our brains do not assign equal relevance to all experiences, and some memories are rapidly forgotten while others endure over time. How does the brain select which experiences are transformed into long-term memories and which ones are deemed reasonable to forget? Memory consolidation refers to the synaptic-cellular and systems-level processes by which newly encoded memory traces are converted into a more stable long-term memory (LTM).^1^^,^^2^ In complex and ever-changing environments, a significant challenge of a memory system is determining which information is meaningful to stabilize as LTM, since information may gain or lose significance over time.^3^ Increasing evidence demonstrates that the longevity of memory is not exclusively determined by the strength of its initial encoding; rather, it is influenced by other mnemonic activities occurring close in time.^4^^,^^5^^,^^6^^,^^7^^,^^8^^,^^9^

The activity-dependent modification of synaptic strength, particularly through long-term potentiation (LTP), is widely proposed as a neural substrate underpinning memory storage. An influential neurobiological model of long-term plasticity and cellular consolidation, the synaptic tagging and capture hypothesis (STC), proposes a mechanism to explain how memories can be stabilized.^10^^,^^11^ In hippocampal slices (but later also demonstrated *in vivo*^12^), a transient form of synaptic potentiation (early LTP or E-LTP), induced by a weak stimulus, can be transformed into a stable and persistent potentiation (late LTP or L-LTP) when shortly preceded or followed by strong stimulation in an independent set of synapses targeting the same neuronal ensemble.^10^^,^^11^ It was proposed that the synapses activated during LTP induction are “tagged” and may “capture” newly synthesized mRNA and proteins (plasticity-related products, PRPs) triggered by stronger neural activity. This process enables the stability and maintenance of LTP and, presumably, LTM.^3^^,^^10^^,^^11^

By extrapolation, the persistence of memory is not exclusively determined by the strength of its initial encoding; instead, a temporal window prevails during which other neural activities may influence cellular consolidation.^3^ Accordingly, the STC model offers a powerful framework for understanding how novelty creates a penumbra of enhanced consolidation for seemingly trivial information that occurs shortly before or afterward.^3^^,^^10^^,^^11^ In this context, the behavioral tagging hypothesis postulated that the formation of LTM involves labeling the sites induced by training, in which the PRPs will be used to establish a memory^5^^,^^13^. These PRPs can be supplied by the same event or by another temporally and spatially associated event.^5^^,^^13^ Numerous sophisticated behavioral studies support a tag-and-capture mechanism whereby weak memories, that would otherwise be forgotten, are “tagged” and rescued by surrounding environmental novelty.^4^^,^^5^^,^^6^^,^^7^^,^^8^^,^^9^^,^^14^^,^^15^^,^^16^^,^^17^^,^^18^^,^^19^^,^^20^ Despite substantial advancements in understanding how weak memories are strengthened, the influence of novelty on the selectivity and structure of long-term memory storage remains not fully understood.

Memory traces for individual events are allocated to a discrete and distributed population of neurons, or ensembles, which are reactivated during later recall.^21^^,^^22^ There is compelling evidence that learning transiently increases the neuronal excitability of an ensemble of neurons, which remain more excitable than their neighbors and may be preferentially biased to be co-allocated to a second event occurring close in time.^22^^,^^23^^,^^24^ By directing information to a shared overlapping neuronal ensemble, memories for separate but related events can be strengthened and linked or integrated, such that recall of one memory increases the likelihood of recalling the other memory (the allocate-to-link hypothesis).^22^^,^^23^^,^^24^ A unique prediction of the STC concept is that two inputs (a weak and a strong input) in temporal proximity converge on the same neurons, allowing the interaction between the tags and the PRPs.^3^^,^^10^^,^^11^ While previous elegant tag-and-capture experiments have demonstrated that memories are co-allocated to an overlapping ensemble in the hippocampus, contributing to the novelty-induced memory enhancement effect,^4^^,^^14^ it remains unclear whether these memories are behaviorally linked to promote consolidation. Although an overlapping ensemble suggests a potential for memory linking, it does not definitively indicate that memories are linked, as the extent of overlap may critically influence whether memories are integrated or remain separate.^25^ Understanding this dynamic is crucial for elucidating how novelty impacts memory consolidation through tag-and-capture mechanisms.

Here, we hypothesized that new information could be linked to a short-lasting memory trace inducing memory consolidation through a tag-and-capture mechanism. By combining behavioral, pharmacological, and cellular imaging approaches, we demonstrated that new information is linked to a weak and transient memory trace, thereby acting as a signal for memory consolidation by engaging an overlapping neuronal ensemble and activating downstream *de novo* protein synthesis in the dorsal CA1 region of the hippocampus (dCA1). Considering that STC and neuronal co-allocation mechanisms in dCA1 are vulnerable to the effects of aging,^14^^,^^22^^,^^26^^,^^27^ we further tested our hypothesis in aged rats. Our results revealed that aging reduced the size of the neuronal ensemble after novelty exposure, decreasing the flexibility of dCA1 neuronal ensembles, and disrupting consolidation. These findings expand our knowledge of how novelty drives memory formation and contribute to refining tag-and-capture models of consolidation.

## Results

### Creating a weak contextual fear memory trace

**Figure 1:**
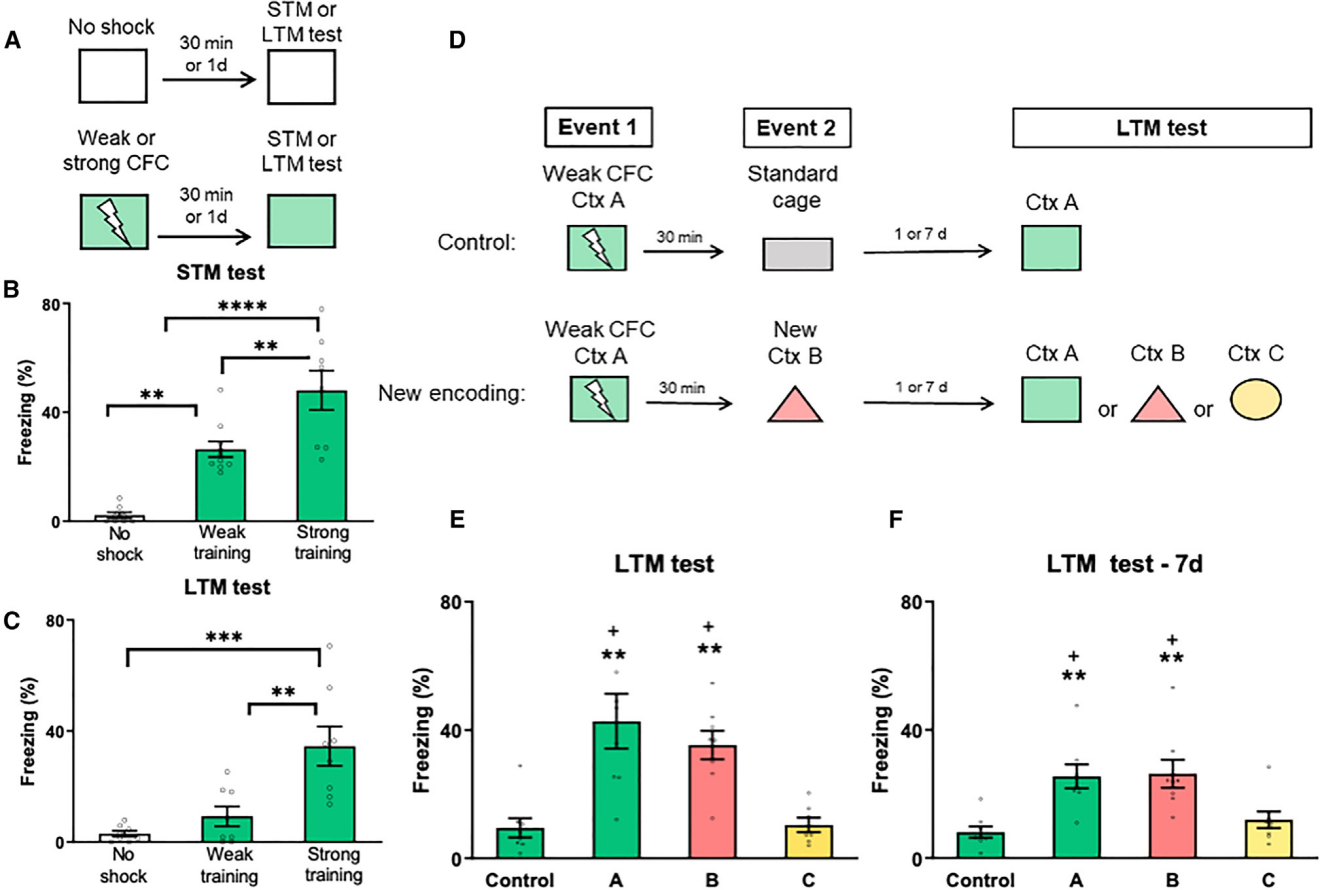
Making memories last: linking new information to a weak memory induces consolidation *Left panel* (A) Schematic representation of the procedure for weak or strong contextual fear conditioning (CFC) training. Freezing levels during short-term memory (STM) (B) or long-term memory (LTM) tests (C). *Right panel* (D) Schematic representation for the experiments depicted at (E and F). Animals underwent events 1 and 2, separated by a 30-min time interval. Event 1 consisted of weak training in context A, and event 2 consisted of exposure to the standard cage (control) or new context (new encoding; New Ctx B). Animals were tested 1 or 7 days later (E) Freezing levels for each group in the LTM test or (F) LTM persistence test. *n* = 7–8 per group. Data are presented as mean ± SEM. and individual data plots. The significance levels are as follows: ∗∗∗∗*p* < 0.0001, ∗∗∗*p* < 0.001, ∗∗*p* < 0.01 compared to the control group; + *p* < 0.05, compared to the group tested in context C, according to one-way ANOVA followed by Bonferroni’s multiple comparisons test, or Kruskal-Wallis followed by Dunn’s multiple comparisons test. Non-significant values are not shown.

To explore whether new information can be linked to a weak memory and mediate consolidation, we initially established a protocol for generating a short-lasting fear memory. Young adult rats underwent training using a standard contextual fear conditioning (CFC) protocol. In this task, animals learn to associate an initially neutral context (the training chamber) with the occurrence of an aversive stimulus (a mild foot shock) and express freezing (an index of fear memory recall) upon their return to the context. CFC is an appropriate behavioral paradigm to assess fear memory transfer to a new contextual event, demonstrating memory linking.^22^ The strength of memory encoding (CFC training) was varied: animals were trained with either 1 or 3 foot shocks (considered weak or strong training, respectively). Following training, animals were tested after 30 min (short-term memory, STM) or 1 day ( LTM), during which freezing behavior was analyzed (Figure 1A). Our results revealed that both weak and strong training promoted the formation of STM, as indicated by significantly higher freezing behavior compared to animals that received no shock (Figure 1B; *n* = 8 per group, one-way ANOVA, F (2, 23) = 25.95, *p* < 0.0001). Strong training led to the establishment of a robust LTM (Figure 1C, *n* = 8 per group; one-way ANOVA, F (2, 21) = 13.22; *p* = 0.0002). In contrast, weak training resulted in the formation of a weak and short-lasting memory that lasted for 30 min (Figure 1B) but which was ordinarily forgotten at 1 day (Figure 1C; *p* > 0.9999).

### Making memories last: linking new information to a weak memory trace induces consolidation

After establishing a weak training protocol, our next step was to investigate whether new information could be linked and rescue the transient memory trace from forgetfulness—a hypothesis that we refer to as novelty-link induced consolidation. We then designed the behavioral paradigm outlined in Figure 1D, using three contexts (namely A, B, and C) with distinct proximal cues (wall color, odor, and texture) (see STAR Methods and *SI Appendix,* Figure S1). These contexts featured transparent front doors that allowed animals a clear view of the surrounding distal cues, creating a common background environment in which separate contextual events could occur. The key idea behind this experimental design was to evaluate whether new contextual information (context B) is linked to the initially weak and short-lasting fear memory (CFC training in context A) and stabilizes the memory trace. If this association occurs, two outcomes would be expected: (1) the linking or transfer of fear between contexts A and B, such that cues from B should trigger the recall of A; and (2) the consolidation of the weak memory, which would otherwise be forgotten. Therefore, during the LTM test, animals should display comparable freezing levels in both linked contexts A and B.

To test our hypothesis, rats were subjected to two events (event 1 and event 2) separated by a 30-min interval. Event 1 involved weak training in context A, which was consistent across all groups, while event 2 consisted of brief 2-min exposure to the new context B (new encoding; New Ctx B). As a control condition, event 2 involved exposure to the standard cage, similar to their home cages, thus representing a familiar box. During event 2, animals exhibited increased time of freezing in context B compared to those in the standard cage, indicating the reactivation of one memory (weak training in Ctx A) while encoding a new one (New Ctx B) (*SI Appendix*, Figure S2A). This memory reactivation mechanism is thought to support the flexible integration of discrete experiences.^28^^,^^29^^,^^30^

The following day, rats underwent LTM testing in contexts A, B, or a novel context C, to assess memory consolidation, memory linking, or overgeneralization, respectively (Figure 1D). As expected, animals exposed to the new encoding event showed increased freezing in context A compared to the Control animals, indicating successful memory consolidation (Figure 1E; *n* = 7–8 per group; Kruskal-Wallis test, H = 18.69; *p* = 0.0053). Importantly, animals subjected to the same event and tested in context B also exhibited increased freezing compared to the control animals, despite never experiencing a shock in that context, demonstrating effective memory linking (*p* = 0.0085). No significant difference in freezing behavior was observed between animals tested in the linked contexts A and B (*p* > 0.9999). Furthermore, this increased freezing cannot be attributed to fear overgeneralization, as animals exhibited a significantly lower freezing level in context C, which they had never encountered before (*p* = 0.0238).

The persistence of memory is tightly dependent on its significance. To determine whether the LTM for the linked contexts is long-lasting, a separated cohort of animals underwent the same procedures aforementioned but was tested 7 days later. Remarkably, LTM for the linked contexts A and B persisted for at least 7 days (Figure 1F; *n* = 8 per group; one-way ANOVA, F (3, 28) = 8.057; *p* = 0.0005). These findings demonstrate that new contextual information is linked to the initially weak, short-lasting memory, leading to long-lasting memories that now persist across days.

This phenomenon resembles tag-and-capture experiments where weak memories are rescued by novelty exposure around the time of encoding.^4^^,^^5^^,^^6^^,^^7^^,^^8^ Similarly, memory consolidation was not dependent on the order of events; it was achieved even when animals explored the new context B before weak encoding in context A, which is analogous to “strong-before-weak” STC protocols^31^ (S*I Appendix*, Figures S3A and S3B). Moreover, as previously shown,^5^^,^^6^ exploration of a familiar context B failed to stabilize STM into LTM (S*I Appendix*, Figures S3C and S3D).

A control experiment was conducted to demonstrate that the link or transfer of fear memory from context A to B was not influenced by environmental geometry or distinct sensory cues, as these contexts are behaviorally equivalent and have no natural significance for animals (*SI Appendix*, Table S1). Additionally, novelty-link induced consolidation depended on previous contextual learning rather than sensitization. This was evident by the absence of memory consolidation when event 1 consisted of an immediate foot shock protocol (*SI Appendix*, Table S1).

### Making memories last: reactivating a weak memory trace induces consolidation

**Figure 2:**
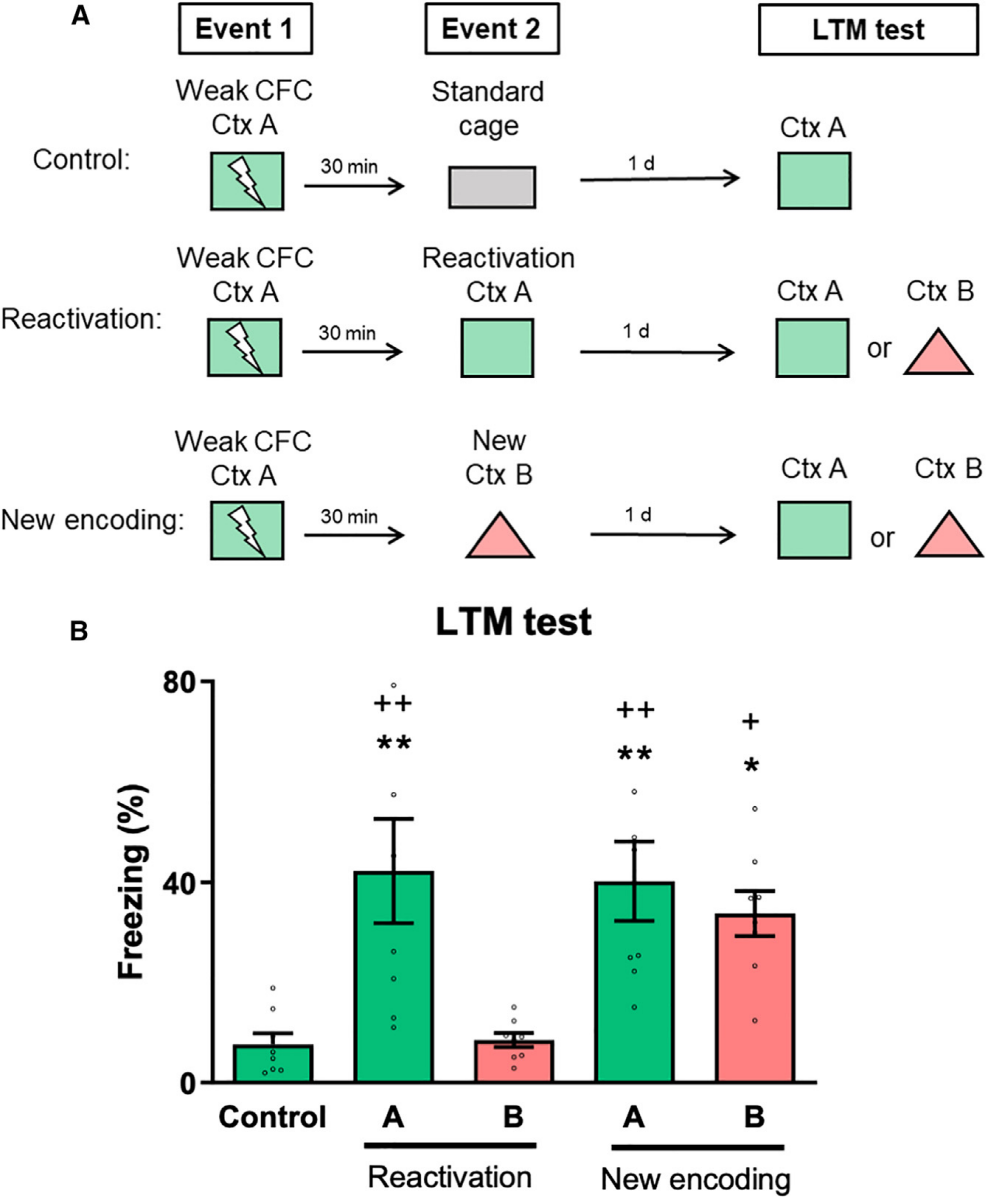
Making memories last: reactivating a weak memory trace induces consolidation (A) Schematic representation for the experiments depicted at B. Animals underwent events 1 and 2 separated by a 30-min time interval. Event 1 consisted of weak training in context A, and event 2 consisted of exposure to the same context (reactivation, Ctx A), the new context (new encoding, New Ctx B), or the standard cage (control). (B) Freezing levels for each group in the LTM test. *n* = 8 per group. Data are presented as mean ± SEM with individual data points plotted. The significance levels are as follows: ∗∗*p* < 0.01 and ∗*p* < 0.05 compared to the Control group; ++*p* < 0.01 and +*p* < 0.05 compared to the group re-exposed to context A and tested in context B, according to one-way ANOVA followed by Bonferroni’s multiple comparisons test. Non-significant values are not shown.

The results aforementioned suggest that encountering new information within a shared environment can trigger the reactivation of weak memory. Following reactivation, memories are thought to enter an active state that enables the weakening (extinction),^32^ strengthening, or modification^33^ of the memory trace. Brief exposure to contexts or cues associated with fear conditioning can strengthen or update memories in the days following encoding through a process known as reconsolidation.^32^ We then examined whether directly reactivating the weak memory trace would induce memory consolidation. Rats underwent event 1 and event 2 separated by 30 min, as previously. In this experiment, event 2 involved exposing the animals to the same context as the training (cues unchanged, but no shock) (reactivation, Ctx A), while other groups were exposed either to the new context B (new encoding, New Ctx B), or to the standard cage (control) (Figure 2A). During event 2, rats exhibited significantly more freezing in context A compared to rats that explored the standard cage or context B, indicating a higher level of memory reactivation in the training context, where the original fear learning occurred (*SI Appendix*, Figure S2B). On the following day, animals were tested for LTM in contexts A or B. Rats subjected to the reactivation or new encoding events spent significantly more time freezing in context A compared to control animals (Figure 2B; *n* = 8 per group; one-way ANOVA, F (4, 35) = 6.754; *p* = 0.0004). Importantly, unlike novelty-link induced consolidation, reactivation of the weak memory generated a context A-specific LTM as animals froze more in context A than in context B (*p* = 0.0026). Collectively, these results indicate that memory updating processes, triggered by the link of new information or the reactivation of the memory trace, can modulate the strength of weak memory, possibly bringing the memory back to an active and malleable state.

### The penumbra of novelty-link induced consolidation: boundary conditions

**Figure 3:**
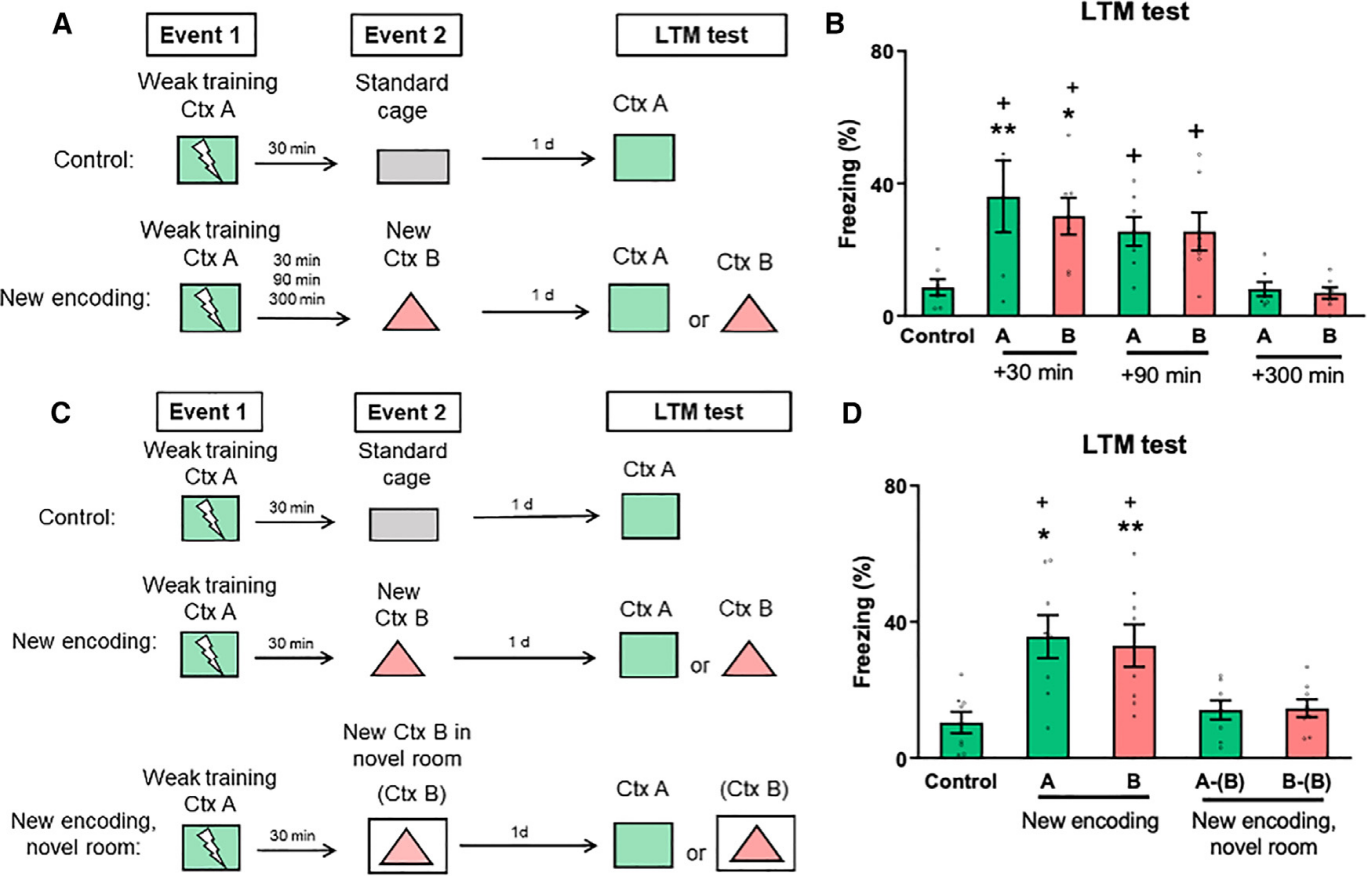
The penumbra of novelty-link induced consolidation: boundary conditions *Top panel* (A) Schematic representation for the experiments depicted in panel B. Animals underwent event 1 and event 2 separated by different time intervals (30, 90, or 300 min). Event 1 consisted of weak training in context A, whereas event 2 consisted of either exposure to context B (new encoding, New Ctx B) or to the standard cage (control). (B) Freezing levels for each group in the LTM test. *Bottom panel* (C) Schematic representation for the experiments depicted at D. Animals underwent event 1 and event 2 separated by a 30-min time interval. Event 1 consisted of weak training in context A. Event 2 consisted of exposure to context B in a separate room (New Ctx B, novel room), in the same room (new encoding, New Ctx B), or to the standard cage (control). (D) Freezing levels for each group in the LTM test. n = 7-8 per group. Data are presented as mean ± S.E.M. and individual data plots. The significance levels are as follows: ∗∗*p* < 0.01, ∗*p* < 0.05, compared to the control group, as determined by one-way ANOVA followed by Bonferroni’s multiple comparisons test (considering time as the factor); + *p* < 0.05, compared to the groups that explored context B 300 minutes later or in a novel room, as determined by two-way ANOVA followed by Bonferroni’s multiple comparisons test (considering time and context as factors). Non-significant values are not shown.

Consistent with the STC predictions, a weak memory can be strengthened by environmental novelty, which occurs in temporal proximity and creates an enhanced penumbra of memory retention.^3^^,^^5^^,^^6^^,^^10^^,^^34^ In allocate-to-link studies, initially neutral contextual memories encoded 300 min apart become linked.^22^ When one of these memories is updated days later by pairing the context with a strong foot shock, the fear response transfers to the other.^22^ To investigate whether there is a temporal window for memory consolidation through linking new information, we arranged the time interval between events 1 and 2 (new encoding, New Ctx B) to be 30, 90, or 300 min apart (Figure 3A). Control rats were exposed to the standard cage 30 min after event 1. In the second event, animals that explored the new context B 90 or 300 min after event 1 exhibited similar freezing levels in the standard cage, indicating that memory was not reactivated and interleaved with the new experience (*S1 Appendix,* Figure S4A). On the subsequent day, we found that delaying the new encoding by 90 and 300 min failed to induce consolidation. Rats tested in contexts A and B exhibited comparable levels of freezing as the control group (Figure 3B; *n* = 7 per group, one-way ANOVA, F (6, 42) = 6.270, *p* = 0.9999). Unlike the previous allocate-to-link study,^22^ the absence of memory linking at 300 min in our experiment is likely due to the weak intensity of training. Additional experiments revealed that weak training, without subsequent exposure to contextual novelty, resulted in STM lasting for 30 min, but not 90 or 300 min (*SI Appendix*, Figures S5A and S5B). Therefore, there is a restricted temporal gap (less than 90 min) within which the weak memory trace may incorporate novel information. These findings confirm the existence of a critical temporal window for novelty-induced memory enhancements, as previously reported ^5^^,^^6^.

Novelty encompasses a broad spectrum, ranging from experiences that can be related to past experiences to those that are completely novel and unrelated to any past experiences.^35^ Distinct hippocampal ensembles are recruited in response to absolute environmental changes.^36^^,^^37^ In principle, if the linking of new information to a weak memory depends on a common neural substrate between memories, then it is expected that an entirely novel event would be encoded separately, and no consolidation would occur. To investigate whether an entirely novel environment influences memory consolidation, rats were subjected to event 1, and 30 min later, received event 2. We then increased the degree of novelty by moving the animals to explore the new context B in a separate room (new encoding, novel room), thereby changing both proximal and distal cues (Figure 3C). Other cohorts of rats were exposed to context B (new encoding) or the standard cage (control) in the same room. During event 2, animals exposed to the new context B in a novel room (New Ctx B, novel room) exhibited similar levels of freezing as animals in the standard cage, indicating a lack of memory reactivation and no interaction with the current new experience (*SI Appendix*, Figure S4B). As expected, on the subsequent day, animals that had explored the new context B in a novel room (New Ctx B, novel room) failed to exhibit fear in contexts A or B (Figure 3D; *n* = 8–9 per group, one-way ANOVA, F (2, 21) = 0.6560; *p* = 0.5292). One possibility is that merely returning to the same spatial context *per se* is sufficient to reactivate the weak memory trace and induce memory consolidation, regardless of the nature of the events. However, this hypothesis was not supported, as other experiments, such as the exploration of the standard cage or a familiar context B in the same room, did not lead to the formation of LTM (*p* = 0.9995). Taken together, the data suggest that the ability to link new information to stabilize a weak memory is constrained by both the duration of the weak memory trace and the degree of novelty experienced by the subject.

### New information is linked to the weak memory trace through an overlapping neuronal ensemble in the dorsal hippocampus

As postulated by the STC concept, weak and strong inputs converge on a common neuronal population.^10^^,^^11^ We then aimed to investigate whether neuronal representations of events 1 and 2, separated by a 30-min interval, are co-allocated to an overlapping neuronal ensemble. Contextual memories are encoded in hippocampal ensembles, ^22^^,^^38^ and several lines of evidence support the molecular, functional, and anatomical heterogeneity of the hippocampus along the dorsoventral axis.^39^^,^^40^^,^^41^ Hippocampal ensembles in the dorsal (dCA1) and ventral CA1 (vCA1) regions have both been implicated in contextual memory.^22^^,^^38^ However, they exhibit distinct activity patterns and are thought to represent different aspects of the experience.^22^^,^^38^ Therefore, we imaged dCA1 and vCA1 neuronal ensembles using cellular compartment analysis of temporal activity by fluorescence *in situ* hybridization (catFISH).^42^^,^^43^ This technique enabled us to detect the expression of two plasticity-related genes, *Homer 1a* (*H1a*) and activity-regulated cytoskeleton-associated protein (*Arc*).^42^^,^^43^ By analyzing the distinct time course of transcription, we were able to distinguish between neuronal ensembles activated by the first event (*H1a*+ neurons, event 1 ensemble), those activated by the second event (*Arc*+ neurons, event 2 ensemble), and those activated by both events (*H1a+/Arc*+ neurons, overlapping ensemble)^42^^,^^43^ (see STAR Methods and *SI Appendix*, Figures S6A and S6B). Comparable numbers of neurons were counted across groups in both dCA1 and vCA1 (*SI Appendix*, Table S2).

**Figure 4:**
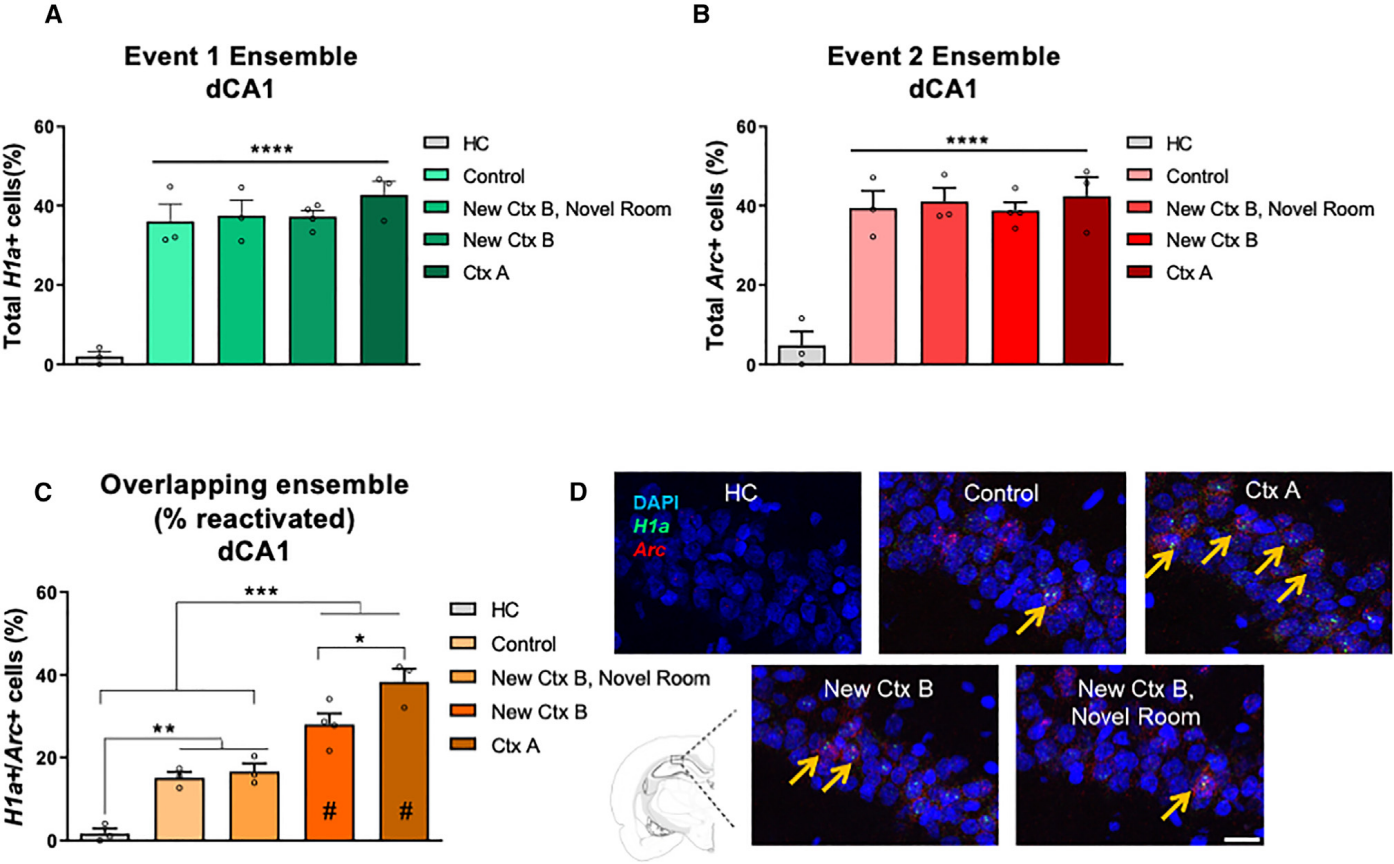
New information is linked to the weak memory trace through an overlapping neuronal ensemble in the dorsal hippocampus (A) Percentage of active neurons during event 1 (total *H1a*+ neurons, ensemble 1) in dCA1. (B) Percentage of active neurons during event 2 (total *Arc*+ neurons, ensemble 2) in dCA1. (C) Percentage of active neurons during events 1 and 2 (*H1a*+/*Arc*+ neurons, overlapping neuronal ensemble) in dCA1. (D) Representative microphotographs of neuronal ensembles in dCA1 for each group. *n* = 3–4 animals per group. Data are presented as mean ± SEM and individual data plots. The significance levels are as follows: ∗∗∗∗*p* < 0.0001, ∗∗∗*p* < 0.001, ∗∗*p* < 0.01, ∗*p* = 0.05, according to one-way ANOVA followed by Bonferroni’s multiple comparisons test. # indicates a significant difference between the percentage of overlap and its chance level value. The *H1a*, *Arc*, and DAPI staining are shown in green, red, and blue, respectively. Orange arrowheads indicate *H1a+/Arc*+ cells. Scale bar, 20 μm. Non-significant values are not shown.

In dCA1 (Figures 4A and 4B), the percentage of neurons active in the home cage group (HC) was very low, whereas there was a significant increase in the proportion of neurons active during events 1 and 2 (total *H1a*+ and total *Arc*+ neurons, respectively) for all groups subjected to the behavioral procedures (∼40% neuronal activation). The observed neuronal activity levels are consistent with previous studies examining behaviorally induced *H1a*/*Arc* expression in dCA1 pyramidal neurons^43^ and *in vivo* imaging methods.^22^ These results are further supported by the counts of *H1a*+ only and *Arc*+ only neurons (*SI Appendix*, Table S2).

The main finding was that activated neuronal ensembles overlapped in a manner sensitive to the degree of environmental novelty (Figures 4C and 4D, *n* = 3–4 animals per group, one-way ANOVA, F (4, 11) = 36.02, *p* < 0.0001). In dCA1, a higher percentage of overlap was observed after memory reactivation (Ctx A), consistent with the notion that exposure to the same environment twice activates the same neuronal ensembles.^43^ Most significantly, the exposure to the new context B (New Ctx B) activated ensembles with an intermediate level of overlap, suggesting a graded neuronal response to sensory changes.^43^ These results, together with our previous behavioral findings, corroborate that reactivation of ensembles during new encoding (neuronal overlapping) supports memory linking,^22^^,^^24^ and serves as a neuronal signature of novelty-induced memory enhancement.^4^^,^^14^

If new information is linked to a weak memory through an overlapping neuronal ensemble in dCA1, we would expect to observe reduced levels of overlap between events that did not promote memory consolidation. Consistent with this hypothesis, the overlap of activated ensembles was lower when animals explored the standard cage (control), or context B in a separate room (New Ctx B, novel room). This reduced overlap aligns with findings that dorsal hippocampal ensembles exhibit minimally overlap after exploration of familiar contexts,^4^ or after changes of locations.^37^^,^^43^^,^^44^ Thus, dCA1 neuronal ensembles are modulated by environmental conditions. When new information is encountered within a common environment as the weak memory, it is more likely to activate an overlapping neuronal ensemble, which may facilitate the interaction between synaptic tags and the PRPs. Previous work has demonstrated that a 5-min exploration of novel floor substrates in a box located in a different room can support memory consolidation for weak appetitive spatial memory.^7^ However, it remains possible that this type of novelty activated a smaller overlapping ensemble in the dCA1, which was still sufficient to modulate PRP synthesis and enhance memory. Further investigation is needed to better understand how different types of novelty and distal cues influence memory formation. Conversely, comparisons among all groups (except HC) revealed similar percentages of overlap within vCA1, suggesting that vCA1 ensembles are less affected by environmental novelty (*SI Appendix*, Figures S7C and S7D). Overall, these suggest that the magnitude of overlap between neuronal representations in dCA1, but not in vCA1, underlies the link of new information to the weak memory.

### New information triggers *de novo* protein synthesis in dCA1 to induce consolidation

**Figure 5:**
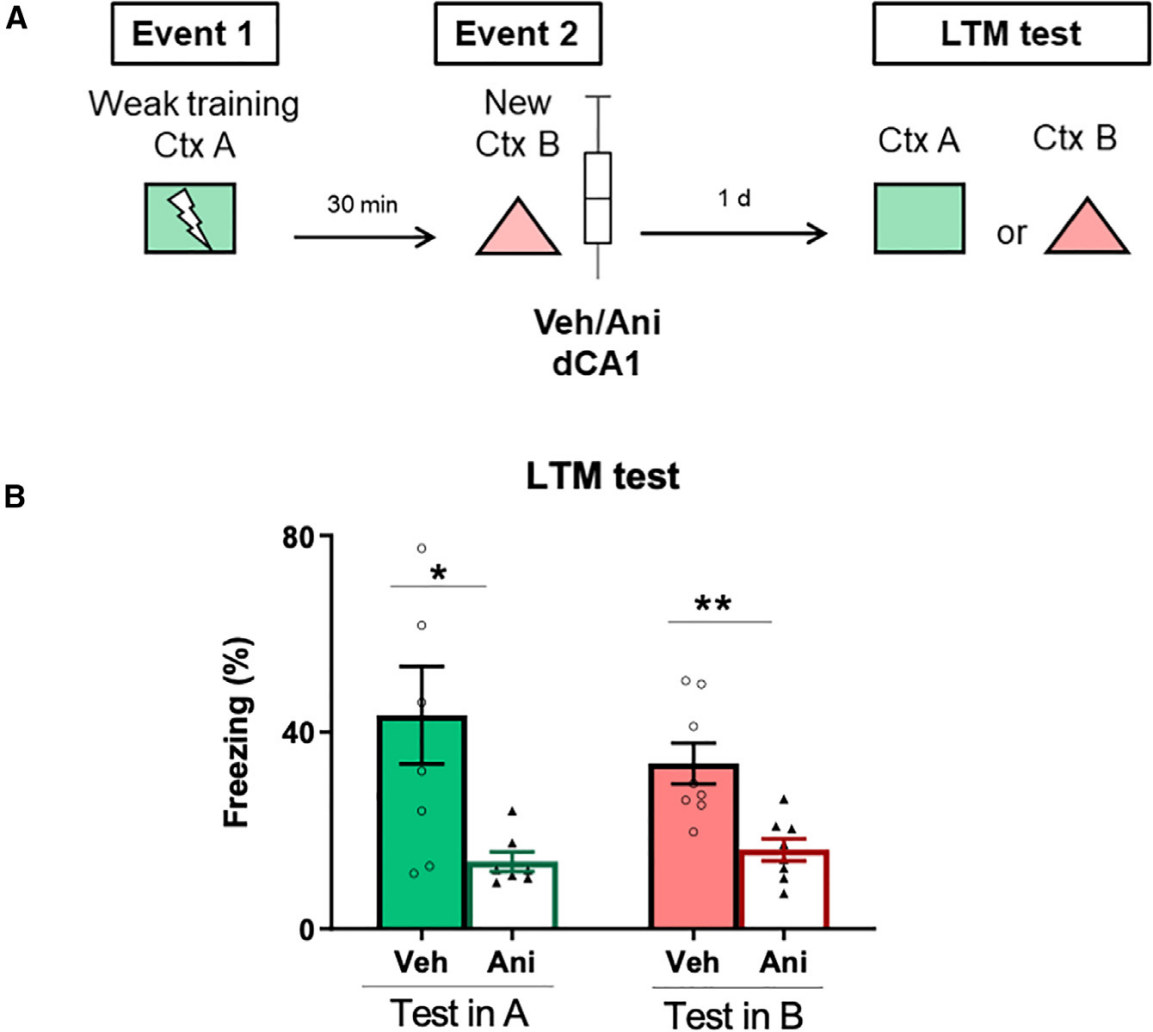
New information triggers *de novo* protein synthesis in dCA1 to induce consolidation (A) Schematic representation of protein synthesis inhibition in dCA1. Animals underwent event 1 and event 2 separated by a 30-min time interval. Event 2 consisted of exposure to context B (new encoding, New Ctx B). Immediately after event 2, animals received either anisomycin (Ani) or vehicle (Veh) in dCA1. (B) Freezing levels in the LTM test. *n* = 7–8 per group. Data are presented as mean ± SEM and individual data plots. The significance levels are as follows: ∗∗*p* < 0.01, ∗*p* < 0.05, according to unpaired t test or Mann-Whitney tests. Non-significant values are not shown.

The hallmark of cellular consolidation is the induction of gene expression and *de novo* protein synthesis (PRPs), which are fundamental requirements for the stabilization of memory traces.^45^^,^^46^^,^^47^ The synthesis of PRPs can be triggered by the encoding itself, when stimuli are sufficiently strong, or by surrounding novel experiences.^3^^,^^5^^,^^6^^,^^10^^,^^16^^,^^18^ To confirm that new information provided newly synthesized proteins to strengthen the weak memory, animals were exposed to events 1 and 2 (New Ctx B) and infused with a protein synthesis inhibitor, anisomycin (Ani), or its vehicle (Veh), in dCA1 immediately after novel context exposure (Figure 5A). The results showed that inhibiting protein synthesis in dCA1 prevented memory consolidation, as the infusion of Ani decreased fear memory in contexts A (*n* = 7–8 per group, two-sided Mann-Whitney test, U = 7, *p* = 0.0140) and B (*n* = 8 per group, two-sided unpaired t test, t = 3.712, df = 14, *p* = 0.0023) (Figure 5B). Hence, exposure to the new context triggers *de novo* protein synthesis in dCA1, inducing memory consolidation.

Given that aging is accompanied by functional alterations in dCA1, including a decreased neuronal overlap between ensembles ^14^^,^^22^^,^^26^ and deficits in the induction of STC in hippocampal slices,^27^ we reasoned that the process of linking new information to stabilize a weak memory trace may be disrupted in aged animals. We explored this possibility in the following text.

### Age-associated deficits in novelty-link induced consolidation

**Figure 6:**
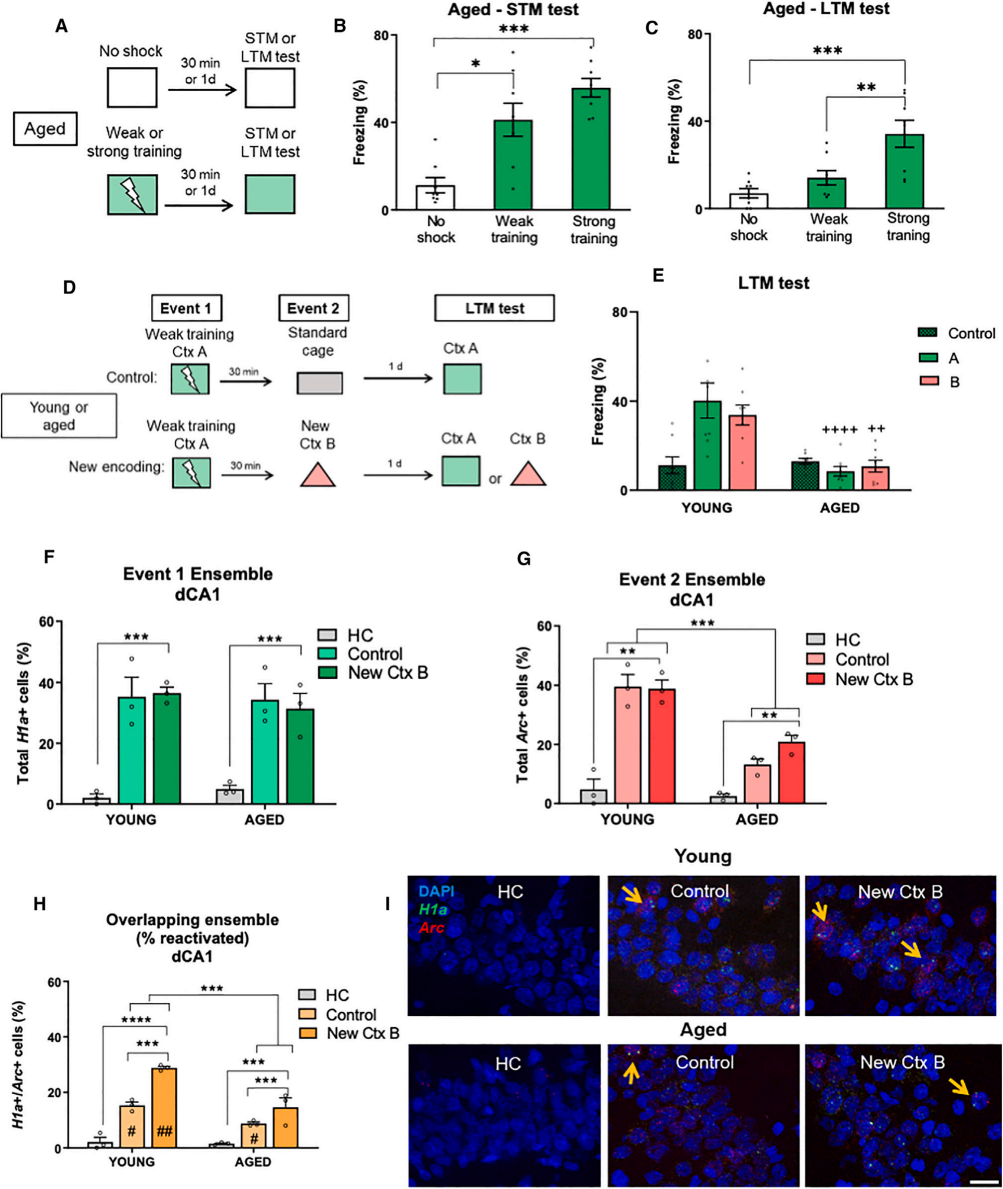
Age-associated deficits in novelty-link induced consolidation *Top panel.* (A) Schematic representation of the procedure for the weak or strong contextual fear conditioning (CFC) training in aged rats. (B–D) Freezing levels in the (B) short-term memory (STM) or (C) long-term memory (LTM) tests. *Middle panel* (D) Schematic representation for the experiments depicted at (E). Young adult or aged animals underwent event 1 and event 2 separated by a 30-min time interval. Event 1 consisted of weak training in context A. Event 2 consisted of exposure to context B (new encoding, New Ctx B) or standard cage (control). Animals were tested for LTM in contexts A or B. (E) Freezing levels in the LTM test. *n* = 8 per group. The significance levels are as follows: ∗∗∗*p* < 0.001, ∗∗*p* < 0.01, according to Kruskal-Wallis or one-way ANOVA followed by Dunns or Bonferroni’s multiple comparisons test, respectively (with group as the factor within the same age). ####*p* < 0.001, compared to the young animals, according to two-way ANOVA followed by Bonferroni’s multiple test (with group and age as factors). *Bottom panel.* catFISH experiments in young and aged animals. (F) Percentage of active neurons during event 1 (total *H1a*+ neurons, ensemble 1) in dCA1. (G) Percentage of active neurons during event 2 (total *Arc*+ neurons, ensemble 2) in dCA1. (H) Percentage of active neurons during events 1 and 2 (*H1a*+/*Arc*+ neurons, overlapping neuronal ensemble) in dCA1. (I) Representative microphotographs of neuronal ensembles in dCA1 for each group. The *H1a*, *Arc*, and DAPI staining are shown in green, red, and blue, respectively. Orange arrows indicate *H1a*+/*Arc*+ cells. Scale bar, 20 μm *n* = 3–4 animals per group. Data are presented as mean ± S.E.M. and individual data plots. The significance levels are as follows: ∗∗∗∗*p* < 0.0001, ∗∗∗*p* < 0.001, ∗∗*p* < 0.01, ∗*p* < 0.05, according to one-way ANOVA followed by Bonferroni’s multiple comparisons test. # indicates a significant difference between the percentage of overlap and its chance level. ##*p* < 0.01, #*p* = 0.05, according to one-sample t test. Non-significant values are not shown.

Aged rats (16–18 months old) underwent training (weak or strong training) and were tested for STM or LTM (Figure 6A). Similar to young adult animals, strong training generated LTM (Figure 6C; *n* = 8 per group, *n* = 8 per group, one-way ANOVA, F (2, 21) = 11.18; *p* = 0.0005) whereas weak training generated an STM (Figure 6B, *n* = 8 per group, one-way ANOVA, F (2, 21) = 16.69, *p* < 0.0001) but not LTM (Figure 6C, *p* = 0.4711). We next investigated how aging affects the link of new information to stabilize a weak memory. Aged and young adult rats were exposed to event 1, and 30 min later, for event 2, they were either exposed to the new context B (new encoding, New Ctx B) or the standard cage (control) (Figure 6D). During event 2, the performance of aged and young animals was indistinguishable, as aged rats also exhibited fear memory reactivation while encoding new information (*SI Appendix*, Figure S8). However, during the LTM test, aged rats tested in contexts A or B exhibited a significantly lower level of freezing compared to young rats (Figure 6E; two-way ANOVA, age: F (1, 42) = 25.55, *p* < 0.0001; contexts: F (2, 42) = 4.707, *p* = 0.0143; interaction: F (2, 42) = 8.244, *p* = 0.0010), and this lower level was similar to that shown by the control animals (*n* = 8 per group, one-way ANOVA, F (2, 21) = 1.120; *p* = 0.3451). Importantly, this age effect is not due to general consolidation impairment, since no LTM age-related deficit was observed when aged animals were trained with a strong protocol (Figure 6C). These results ratify that peri-encoding novelty exposure is ineffective in aged rats, as previously demonstrated in spatial memory tasks.^14^^,^^48^^,^^49^

To gain deeper insights into the cellular mechanisms underlying the novelty-link consolidation deficit, we conducted imaging of neuronal ensembles in the aged hippocampus. In dCA1, similar proportions of neurons were activated in both young and aged rats during event 1 (total *H1a+* neurons, event 1 ensemble) (Figure 6F; *n* = 3 per group; two-way ANOVA, age: F (1, 12) = 0.1236, *p* = 0.7313; groups: F (2, 12) = 37.38, *p* < 0.0001; interaction: F (2, 12) = 0.5348, *p* = 0.5991). Despite a comparable number of neurons counted in both aged and young adult animals (*SI Appendix*, Table S2), there were significant age-related changes in the activation patterns and overlap between neuronal ensembles. During event 2, the reduced number of active neurons (total *Arc*+ neurons, event 2 ensemble) in the dCA1 of aged rats compared to young rats indicates decreased post-novelty neuronal activity (Figure 6G; *n* = 3 per group; two-way ANOVA, age: F (1, 12) = 30.59; *p* = 0.0001; groups: F (2, 12) = 35.29, *p* < 0.0001; interaction: F (2, 12) = 4.535, *p* = 0.0341). Remarkably, there was a significant reduction in neuronal overlap in dCA1 of aged rats, consistent with findings from prior studies ^14^^,^^22^^,^^26^ (Figures 6H and 6I; *n* = 3 per group; two-way ANOVA, age: F (1, 12) = 41.25, *p* < 0.0001; groups: F (2, 12) = 114.9, *p* < 0.0001; interaction: F (2, 12) = 11.94, *p* = 0.0014). No age-related changes in the number of active neurons or overlap were observed in vCA1 (*SI Appendix*, Figures S9A–S9D), indicating a region-specific vulnerability of dCA1 neurons to aging effects. In summary, these findings reinforce the importance of neuronal ensemble overlap in dCA1 for linking new information to stabilize a weak memory. The decrease in overlap between ensembles in aged rats suggests that this reduction may contribute, at least partially, to the deficit in novelty-link induced consolidation.

## Discussion

Our findings confirm and extend previous evidence indicating that the longevity of memory is influenced by other novel and/or unexpected events occurring nearby, which tag and rescue weak memory traces, as predicted by the STC model.^4^^,^^5^^,^^6^^,^^7^^,^^8^^,^^9^ Several key observations emerged from this study. Firstly, our results elucidate how new information can interact with an existing short-lasting memory to enhance its stability and longevity. By linking a new experience to a weak memory, the brain prioritizes and strengthens information that might otherwise be forgotten, thus enhancing the overall richness and persistence of memory representations. Secondly, we identified that this flexible interaction between memories operates within a narrow time window and is driven by the contextual relatedness of memories to be linked. Thirdly, the underlying cellular and molecular mechanisms involve the magnitude of overlap between neuronal representations and *de novo* protein synthesis in dCA1, supporting a tag-and-capture mechanism. Lastly, unlike their younger counterparts, aged animals exhibited reduced activation of neuronal ensembles in response to novelty and reduced neuronal overlap between memories in dCA1, contributing to the age-related failure of consolidation. Together, these findings hold significance for the functional implications of the STC model in memory consolidation, as discussed in the following text.

The STC concept provides a fundamental neurobiological basis for understanding cellular consolidation^3^^,^^10^^,^^11^^,^^12^^,^^34^ and synaptic allocation.^23^^,^^50^ This concept was initially devised to explain input specificity during L-LTP, since *de novo* protein synthesis, critical for L-LTP and LTM, primarily occurs in the cell body. However long-term synaptic potentiation is restricted to recently activated synapses.^11^ It postulates that the persistence of synaptic strength and memory involves the local setting of synaptic tags at glutamatergic synapses in a manner independent of protein synthesis, along with the heterosynaptic modulatory-dependent synthesis and distribution of PRPs induced by other stronger neural activity—triggered simultaneously or not.^3^^,^^34^^,^^51^ When the PRPs are distributed while the tags are temporarily active, tagged synapses may capture the PRPs to stabilize synaptic changes and the memory trace. This implies that the maintenance of hippocampal LTP and memory is sensitive to post-encoding events that likely influence the selectivity of cellular consolidation. Novelty is thought to play a crucial role in cellular consolidation, as predicted by the STC model, creating a “grace period” of enhanced consolidation for seemingly trivial details occurring close in time.^3^^,^^10^ Novel experiences have been shown to activate modulatory systems, leading to the release of dopamine and the upregulation in the synthesis of PRPs in the hippocampus, particularly via a recently characterized locus coeruleus and dCA1 dopaminergic circuit.^8^^,^^15^^,^^18^ Therefore, novelty shortly before or after weak encoding could potentially transform a weak memory into a more enduring one.^34^ This mechanism provides insights into the formation of flashbulb memories,^52^ wherein novel and arousing events enhance the retention of numerous surrounding details and mundane information occurring in temporal proximity that would otherwise be forgotten.^10^

A substantial body of research has confirmed that novel and/or other salient experiences modulate *de novo* protein synthesis and enhance consolidation for other independent weak memories encoded close in time through temporal association.^4^^,^^5^^,^^6^^,^^7^^,^^8^^,^^9^^,^^15^^,^^16^^,^^18^^,^^19^^,^^53^ These studies, considering the behavioral, temporal, and molecular properties, have been interpreted in light of the STC model. Our findings strongly align with the defining features of a tag-and-capture mechanism, supporting our interpretation of the data within the framework of the STC concept. Furthermore, we extend these earlier reports to demonstrate that an initially weak memory, which holds little significance at the time of encoding, acquires greater meaning when relevant new information is incorporated into the weak memory trace. This novelty-link induced consolidation leads to long-lasting memories that are recalled together.

An elegant tag-and-capture experiment has demonstrated that artificially silencing the novelty-related ensemble results in the impairment of LTM recall for a previously weak object recognition memory, indirectly suggesting a linkage between memories for novelty-induced memory enhancement.^4^ Regarding the interaction between a weak and a novel memory trace, our study unveils that memories are linked at the behavioral level for a tag-and-capture mechanism to occur. The existence of such a phenomenon raises questions about its functional relevance. From an adaptive perspective, it may represent a sophisticated mechanism for adaptive behavior and cognitive flexibility. Remembering isolated information often proves insufficient to guide future behavior. Linking new information to existing memories enhances their predictive value and relevance in dynamic environments, allowing individuals to draw upon a broader array of experiences and knowledge when making decisions or responding to similar situations in the future.^23^ Consequentially, weak memories that successfully incorporate new information become enriched and updated, thus they are selectively retained and more resistant to forgetting. In contrast, weak memories that fail to incorporate new information become outdated and are eventually forgotten. Our results, therefore, converge with theoretical proposals suggesting that STC mechanisms play a crucial role in orchestrating and optimally storing multiple memories, facilitating their flexible expression.^22^^,^^50^

What defines a sufficiently “strong” event for modulating other weak memories? Guided by the STC concept, a “strong” event should engage common neural substrates and trigger sufficient upregulation of PRPs to stabilize both pathways.^11^ In our study, brief exposure to a new context served as an effective modulatory event for a weak contextual fear memory by engaging an overlapping ensemble and triggering *de novo* protein synthesis in dCA1. As mentioned earlier, there are distinct types of novel experiences. One commonly studied form of environmental novelty involves the exploration of a novel open field, which has been shown to enhance memory consolidation for weak inhibitory avoidance,^6^ contextual fear, and spatial object recognition memories.^5^ However, it is noteworthy that exposure to a novel open field does not consistently lead to memory enhancements,^49^ and may even impair other memories encoded in close proximity,^54^ possibly due to competition for available PRPs. Other novel experiences include the exploration of novel floor substrates in a box, a procedure that rescued weak spatial memory,^7^^,^^8^^,^^14^^,^^48^^,^^55^ whereas the exploration of novel objects was insufficient to do so.^55^ Moreover, re-exposure to the training environment,^48^ rewarding, and aversive learning experiences have also been shown to modulate weak memories in rodents^55^ and humans.^9^^,^^19^^,^^56^ It becomes evident that other behaviorally salient experiences beyond novelty exploration influence the selectivity of memory consolidation and that not all types of novelty are of sufficient importance to enhance the retention of inconsequential memories.

Novelty signals originating from the hippocampus may be combined with information regarding salience from extrahippocampal structures to reflect the overall significance of the information^57^ and determine whether memory for other events is enhanced. Certainly, the neural circuits activated by novelty, such as the hippocampal-midbrain loops, are coincidentally activated by motivation and salience.^57^ Considering our experimental settings and results, exposure to the new context is perceived by the subject as “behaviorally relevant” because of its relationship with the weak fear memory. This is evidenced by the fear behavioral response to the new context, indicating animals perceived this environment as salient or potentially threatening. When new information shares contextual similarities, such as common environment cues, animals detect the relations between experiences resulting in memory reactivation and integration. Previous tag-and-capture studies in humans demonstrate that rewarding^56^ or punishment events^9^^,^^19^ retroactively enhance memories for conceptually related items but do not generalize to other unrelated items encoded close in time. Therefore, the tag-and-capture mechanism selectively prioritizes memories to be remembered based on their temporal, contextual, and conceptual relations with other salient information. We propose that behaviorally salient and arousal-inducing experiences control the entry of information into long-term storage in a manner consistent with the principles of the STC concept. Novelty, in this context, serves as one of the factors contributing to behavioral salience and arousal, but it is not the exclusive or main determinant. Instead, it is the significance and relevance of the experience to the subject’s prior knowledge that create a penumbra of memory retention for other temporally proximal events. The complex interactions between discrete memories are still beginning to be unveiled.

To date, there is very limited evidence on how aging affects tag-and-capture mechanisms. In aged hippocampal slices, CA1 Schaffer collateral synapses fail to induce STC, as demonstrated by the inability of strong L-LTP inducing stimulation to strengthen a weak E-LTP in two-pathway experiments.^27^^,^^58^ At the behavioral level, our findings, along with those of other studies, have shown that novelty exposure does not facilitate memory consolidation for weakly encoded contextual, spatial object recognition^49^ or spatial rewarding memories.^14^^,^^48^ Our study further extends these findings by demonstrating that aged rats are unable to reactivate a dCA1 neuronal ensemble of comparable size to that of younger rats when exposed to a new context. Since successful novelty-induced consolidation relies on the magnitude of overlap between memory-representing ensembles in the young dCA1, the reduced proportion of reactivated dCA1 neurons (*Arc*+ neurons) in the aged hippocampus may significantly contribute to the failure of tag-and-capture processes.

Previously, Gros and collaborators demonstrated that early aging (8.5–13 months) significantly reduces the size of the encoding-activated neuronal population (*H1a+* neurons) in a weak spatial rewarding task, even more so than the novelty-activated ensemble observed after exploration of novel floor substrates in a box (*Arc+* neurons). This reduction was associated with decreased overlap in the dCA1 region and the subsequent failure of memory consolidation.^14^ This behavioral and cellular evidence suggests that early aging primarily affects tagging during memory encoding, rather than the synthesis or distribution of PRPs.^14^ However, early aging has also been shown to disrupt spatial memory encoding, regardless of training strength (weak or strong).^48^ According to the STC concept and as suggested by the Behavioral Tagging hypothesis, memory encoding is influenced by events occurring at the time of encoding, but the retention or forgetting of memory traces also depends on additional factors, including peri-encoding modulatory events that regulate PRP synthesis.^3^^,^^5^^,^^10^^,^^11^ Therefore, if the process of setting learning tags during spatial memory is impaired, it becomes difficult to distinguish between the effects of aging on encoding and its impact on the modulatory aspects of consolidation, as highlighted in our study.

By leveraging the intact contextual fear encoding during aging, we observed a specific reduction in both the size of the novelty-related ensemble (total *Arc*+ neurons) and its overlap. This suggests that aging affects the synthesis and/or capture of PRPs triggered by the modulatory event while tag-setting remains preserved. Our results are consistent with *in vitro* studies, showing that aged neuronal networks exhibit a reduced pool of PRPs during the induction of long-term plasticity.^58^ This impairment affects late-associative plasticity mechanisms including STC, leading to increased competition for the limited available PRPs, which are captured by strongly potentiated synapses but not by weakly potentiated ones.^58^ It is plausible that tag-setting for spatial memory may be more susceptible to aging or decay more rapidly than for fear memories, suggesting differential effects of aging on tagging or capture mechanisms based on the type of information. Nonetheless, the decrease in the novelty-related ensemble size that we observed may reflect the reduced neuronal excitability of dCA1 neurons after learning in the aged brain,^22^^,^^26^^,^^59^ which could significantly influence where and how subsequent experiences are encoded.

In allocate-to-link studies, aged mice exhibit reduced neuronal overlap between ensembles representing two contextual memories encoded 5 h apart, in contrast to young mice, resulting in an impaired ability to link memories over time.^22^ Artificially enhancing dCA1 neuronal excitability in the aged hippocampus before encoding increases neuronal overlap and rescues this memory linking deficit, underscoring the critical role of intrinsic excitability in these processes.^22^ Recent findings indicate a post-learning ensemble-specific reduction in neuronal excitability within aged dCA1 neurons, which correlates with impaired hippocampal memory consolidation.^26^ Building on these insights, neurons activated during weak encoding (*H1a*+ neurons) may exhibit reduced post-learning excitability, making them less likely to reactivate following novelty exposure (*Arc*+ neurons). This ultimately results in both a smaller novelty-related ensemble size and diminished overlap between ensembles. The reduced levels of neuronal activity and overlapping populations are insufficient to support effective memory consolidation through the linking of new information.

While our convergent behavioral and cellular findings suggest a critical role for the reactivation of dCA1 ensembles in modulating memory consolidation, additional mechanisms likely regulate this process and contribute to the tag-and-capture deficits during aging, including aberrant *Arc* expression. *Arc* is an effector immediate early gene essential for persistent forms of activity-dependent synaptic plasticity and memory (reviewed in the study by Shepherd J.D. and Bear M.F.^60^). *Arc* expression is rapidly and transiently induced by neuronal activation. Its mRNA is enriched in dendrites and targeted to recently activated glutamatergic synapses for local translation.^60^^,^^61^ This synapse-specific feature suggests a role for *Arc* in linking synaptic activity to protein synthesis-dependent synaptic plasticity, consistent with the STC model and supporting *Arc* as a potential PRP candidate.^62^ *Arc* has been shown to be essential for L-LTP and LTM formation, although it is not required for E-LTP and STM.^63^ Given that hippocampal neurons expressing *Arc* mRNA also express Arc protein,^64^ our results suggest that *de novo* protein synthesis is also affected during aging.

Considering the versatile role of *Arc* in coupling neuronal activity patterns to enduring forms of synaptic plasticity, the reduced proportions of *Arc*+ dCA1 neurons in the aged brain following new context exposure indicate that long-term information storage in active networks is impaired in the aged hippocampus, leading to insufficient memory consolidation. While these findings may seem inconsistent with other reports that did not detect age-related changes in the proportions of *Arc*-expressing dCA1 neurons,^65^^,^^66^ they did observe a significant reduction in transcription levels (the amount of mRNA produced per neuron) following exploratory behaviors.^65^ It is important to consider the significant experimental differences between our study and these others. Specifically, they compared middle-aged animals (average age 11.5 months) with senescent animals (average age 24.1 months), so their findings may not directly contradict the results reported here. Overall, the dysregulation of *Arc* may play a pivotal role in cognitive aging and the decline of LTM,^65^ making it a potential target for therapeutic interventions aimed at preventing or rescuing deficits in novelty-link induced consolidation. Furthermore, modulatory brain systems that are activated by environmental novelty, such as the locus coeruleus,^8^^,^^15^ are often affected by aging,^67^ which further strengthens that the synthesis of PRPs is compromised in the aged hippocampus. The relevance of understanding and identifying memory decline mechanisms during aging cannot be overstated.

In light of our findings, we propose an updated and complementary mechanistic framework for understanding how novelty influences memory consolidation. Initially, a weak and short-lasting memory is temporarily allocated to a specific neuronal ensemble in dCA1. In the presence of relevant new information, encoded close in time, dCA1 signals that there is information to gather and promotes the reactivation of the neuronal ensemble. This increased period of plasticity enables the incorporation of new information into the existing memory representation. The overlap between neuronal ensembles allows that PRPs synthesized in response to the new information to be “captured” by eligible “tagged synapses’, resulting in memory linking and stabilization. Consolidation through memory linking depends on the magnitude of overlap between dCA1 neuronal ensembles; if the overlap is too small, it is insufficient to link memories and facilitate the sharing of PRPs for consolidation. Conversely, completely unrelated information is less suitable for reactivating the neuronal ensemble and is allocated as a separate memory. The reduced level of neuronal overlap leads to suboptimal interaction between the tags and PRPs, resulting in the isolation and, ultimately, forgetting of the weak memory trace.

The high degree of hippocampal plasticity and malleability of memory consolidation decays across the lifespan, as new information that might be linked and strengthen another one is unable to do so due to altered patterns of neuronal activity in dCA1 in the aged brain. A remaining puzzle is whether age-related reductions in neuronal activation and neuronal overlap seen here are the cause of consolidation failure and not just a correlate. It would be interesting to test whether increasing neuronal excitability or *Arc* expression in dCA1 neurons in aged animals might help restore this deficit. Taken together, our findings strongly suggest that tag-and-capture and neuronal allocation mechanisms in dCA1 act synergistically to support novelty-link induced consolidation. Further research is still needed to obtain direct evidence and to fully elucidate how STC mechanisms operate at the level of individual spines and synapses in freely behaving animals during memory formation.

In conclusion, our findings highlight the intricate dynamics through which memories influence each other and contribute to memory consolidation. Our proposed framework provides a valuable model for understanding how novelty influences memory consolidation, offering insights that could be leveraged to improve therapeutic approaches for memory-related disorders or applied in educational settings. Dysfunction in tag-and-capture mechanisms is likely a key contributor to a range of cognitive deficits. Given the loss of contextual episodic details during aging,^68^ we speculate that alterations of tag-and-capture mechanisms, as depicted here, underlie this decline in memory function. While the incorporation of new information to stabilize a weak memory confers adaptative advantages, its dysregulation could lead to maladaptive behaviors, including anxiety disorders. For example, the formation of intrusive memories often involves trivial details that become part of the memory and act as reminder cues, or “warning signals”, triggering debilitating symptoms in patients with post-traumatic stress disorder (PTSD).^69^ Future research is needed to explore whether tag-and-capture mechanisms contribute to such conditions and to further understand their role in memory disorders.

### Limitations of the study

Although our behavioral, pharmacological, and cellular approaches strongly mirror critical defining features and predictions of the STC concept, we acknowledge that they do not provide direct evidence. Therefore, we adopted the term “tag-and-capture” in the discussion of our results, recognizing that we did not employ methods to monitor and manipulate individual spines and synapses directly. To fully explore the role of STC mechanisms in novelty-induced consolidation, more direct real-time methods, such as *in vivo* calcium imaging combined with electrophysiological recordings, are essential future approaches.

Another limitation of our study pertains to potential sex differences. We initially tested our hypotheses using male rats due to the sexually dimorphic nature of contextual fear conditioning. Female rodents typically exhibit higher freezing levels and greater fear generalization, which is associated with preferential recruitment of the basal amygdala and reduced activation of the dorsal hippocampus.^70^ We acknowledge that conducting experiments in female rats is critical for enhancing our understanding of the novelty-induced consolidation phenomenon and its clinical and translational value. Expanding our research to include both sexes will enhance our comprehension of novelty-induced consolidation and its broader implications.

## Resource availability

### Lead contact

Further information and requests for resources and reagents should be directed to and will be fulfilled by the lead contact, Suzete Maria Cerutti (smcerutti@unifesp.br).

### Materials availability

This study did not generate new unique reagents.

### Data and code availability

• The data of this study are available from the corresponding author upon request.
• This paper does not report original codes.
• Any additional information required to reanalyze the data reported in this paper is available from the lead contact upon request.

## Acknowledgments

This work was supported by grants from the São Paulo State Research Foundation (FAPESP) (grant 2019/24614-4 to S.M.C.). A.G.S. is a scholar from the Coordenação de Aperfeiçoamento de Pessoal de Nível Superior - Brasil (CAPES) –Finance Code 001. Figures were created with SciDraw (scidraw.io).

## Author contributions

A.G.S. and S.M.C. conceived and designed the research. A.G.S., J.S.B., and B.G.M. conducted the experiments. A.G.S. analyzed the data. L.A.C. contributed new materials. A.G.S. and S.M.C. wrote the manuscript. S.M.C. supervised the study and provided financial support.

## Declaration of interests

The authors declare no competing interests.

## STAR★Methods

### Key resources table

**Table:** 

REAGENT or RESOURCE	SOURCE	IDENTIFIER
**Chemicals, peptides, and recombinant proteins**		

Anisomycin	Sigma-Aldrich	Cat.No.A9789

**Critical commercial assays**		

RNAscope® Fluorescent Multiplex Assay	Advanced Cell Diagnostics, ACDbio	Cat. No.323133
*Arc* target probe	Advanced Cell Diagnostics, ACDbio	Cat. No.317071-C2
*H1a* target probe	Advanced Cell Diagnostics, ACDbio	Cat. No. 433261

**Experimental models: Organisms/strains**		

Rat: Wistar	Center of Experimental Models for Medicine and Biology, Federal University of Sao Paulo	N/A

**Software and algorithms**		

Refor II	Insight, Brazil	EP162
ANY-Maze version 7.1	Stoelting Co., USA	60000
GraphPad Prism version 8.0	GraphPad Software, San Diego, USA	N/A
ImageJ/Fiji	NIH, Bethesda, MD	https://imagej.nih.gov/ij/

### EXPERIMENTAL MODEL AND STUDY PARTICIPANT DETAILS

#### Animals

All procedures were conducted in compliance with the guidelines set by the National Institutes of Health Guide for the Care and Use of Nonhuman Animals in Research and were approved by the local Committee Governing the Ethics on the use of Animal Experimentation of the Federal University of São Paulo (Approval number 3215141119). Experiments were conducted on young adults (3–6 months) or aged (15–18 months) male Wistar rats obtained from the Center of Experimental Models for Medicine and Biology (CEDEME – UNIFESP). All experiments were carried out during the light period of the day (between 8:00 a.m. and 14:00 p.m.). Animals were socially housed with 2–4 littermates per cage, with unrestricted access to food and water. Subjects were randomly assigned to the different experiments and conditions. Researchers were aware of the experimental group assignment during the training and testing of all animals but were blinded during offline analysis.

### Method details

#### Contextual fear conditioning (CFC) apparatus

The original CFC apparatus consisted of two conditioning chambers (32 × 27 × 26 cm; Insight, Brazil) with clear Plexiglas front doors and ceilings, aluminum side walls, and grid floors (18 bars with 0.3 mm in diameter) wired to a shock generator controlled by Refor II software (Insight, Brazil). Different contexts were designed for the experiments, each with distinct visual, tactile, and olfactory cues. Context A featured the original chamber configuration, dim white light, and a scent of 20% ethanol. Context B had a triangular roof, plywood floor and walls with black and white stripes, red LED lighting, and a solution of 1% acetic acid placed underneath the chamber to provide a distinct olfactory cue. Context C featured a black semi-circular plastic wall, lights off, and a grid floor overlaid with black plastic and was scented with 1% vanilla essence. Contexts B and C were counterbalanced across animals in within-context experiments. The contexts were cleaned with 70% ethanol before and between runs. Throughout the experiments, because of the Plexiglass front doors, the rats could see all available distal cues in the room, such as the stand holding the recording equipment and walls.

#### Handling

To reduce stress, animals underwent individual habituation to handling by the researcher, with sessions lasting 5 minutes each. Handling sessions occurred in the animal housing room. On the training and testing days, animals were transported in their home cages to an anteroom 1 hour before the experiment and were left undisturbed. After this period, animals were individually transported to the experimental room in plastic maintenance cages containing bedding identical to their home cages.

##### Event 1

Animals underwent fear conditioning using a standard CFC paradigm in Context A. For weak training experiments, animals were habituated to the context for 2 minutes followed by a single foot shock (0.3 mA for 0.5 seconds). Animals remained in the context for an additional 1 minute after shock and then were placed back in the maintenance cage in the anteroom. For strong training experiments, animals were habituated to Context A for 2 minutes followed by three foot shocks (0.3 mA for 0.5 seconds each) delivered at 121 seconds, 151 seconds, and 181 seconds. After the last shock, animals remained in the context for 1 additional minute before being returned to the maintenance cage. In the immediate shock protocol, animals were placed in Context A, received an immediate shock, and were removed from the context.

##### Event 2

Event 2 involved different behavioral conditions depending on the experiment. Animals were exposed to a new context (referred to as New Ctx B) for 2 minutes, either before or after training (event 1), at different time intervals. As the Control condition, rats explored a white plastic cage (16 × 34 × 42 cm) with bedding, identical to their home cages (standard cage) for 2 minutes in the anteroom. Alternatively, they were exposed to the Context A, the same context as the training (referred to as Ctx A), for 2 minutes. Context B was located either in the same room as Context A or in a separate room, as specified in the experiments. No shock was delivered.

#### Testing

Testing sessions lasted 5 minutes, with conditions mirroring the training, except for the absence of shock. STM tests were conducted 30 minutes after training, while LTM tests were conducted either 1 or 7 days after training. Testing sessions occurred in Context A, where training took place, or in Context B. In certain experiments, testing was conducted in a novel context presented only during the test (Context C). At the end of the tests, animals were returned to their home cages and transported back to the animal facility room.

#### Stereotaxic surgery and cannula implantation

For the experiment involving hippocampal protein synthesis inhibition, animals were anesthetized with a ketamine (100 mg/kg), xylazine (10 mg/kg) and acepromazine (1 mg/kg) intraperitoneal injection and placed into a stereotaxic head frame (Insight Equipment LTDA, Brazil) on a heating pad. A craniotomy was performed with a small drill and guide cannulas (24-gauge; 12 mm length) were implanted bilaterally targeting the dCA1 (AP -3.8 mm, ML ± 2.5 mm from bregma, DV -2.5 mm).^71^ The cannulas were anchored to the skull with two miniature stainless-steel screws and multiple layers of dental cement for a head cap. Mandrills were inserted into the guide cannulas to prevent clogging. Following surgery, animals received physiological saline (10 mL/kg, subcutaneously) and meloxicam (0.2 mg/kg, subcutaneously) for postoperative hydration and analgesia. Animals were individually housed to prevent cannula damage and were given a recovery period of 7 days. During this period, animals were monitored for evaluation of pain/distress. Cannula placement was verified postmortem by histology, and this was used as a criterion for the exclusion of animals (*n* = 2). After testing, animals were euthanized, and their brains were rapidly extracted and stored at −20°C until sectioning. Coronal sections of 30 μm thickness were cut using a cryostat, mounted onto gel-coated slides, and examined to confirm the position of the cannulas in the hippocampal tissue under an optical microscope (*SI Appendix*, Figure S10). Only animals with correct cannula placement were included in the behavioral analysis.

#### Drug preparation and microinjection

Anisomycin is a potent and reversible inhibitor of protein synthesis.^4^^,^^5^^,^^6^^,^^7^ Anisomycin (Sigma-Aldrich, catalog #A9789) was dissolved in 1 N HCl, diluted with saline in 10× PBS, and adjusted to pH 7.4 by adding 1 N NaOH for a final concentration of 125 μg/μ.^7^ Aliquots were stored at −20°C until further use.^7^ For the infusion procedures, animals were gently restrained with a towel in their maintenance cages and injection cannulas were placed into the guide cannulas protruding 1 mm below the tip. A total volume of 1 μL of anisomycin or vehicle (PBS) was injected into the dCA1 at a flow rate of 0.25 μL/min using an automatic infusion pump (Model Bi2000 - Insight Equipment LTDA, Brazil). The injection cannulas remained in the brain for 1 additional minute after infusion and were then slowly withdrawn to prevent backflow. The dose and volume were chosen based on literature data showing the effects of anisomycin on memory consolidation.^7^

#### catFISH experiments

The catFISH (cellular compartment analysis of temporal activity by fluorescence *in situ* hybridization) is a cellular imaging method that allows for the detection of differences in neuronal activity and overlap between neuronal ensembles across multiple brain regions in the same animals.^42^ Given the differential time course of transcription and subcellular localization of *Arc* and *H1a* mRNA, it is possible to infer whether a neuronal ensemble was active during two discrete events at different time points.^42^ *Arc* mRNA is generated from a short transcript (∼3.5 kb), whereas *H1a* mRNA is generated from a long transcript (∼45 kb). Upon neuronal activation, *Arc* mRNA is rapidly expressed in the nuclei (within 2 minutes after its induction), and then transported to the cytoplasm (25–35 minutes), whereas *H1a* mRNA is co-expressed in the nuclei after 25–40 minutes. Consequently, neurons expressing *Arc* mRNA were active in the preceding 0–8 minutes (event 2+ neurons) while neurons expressing *H1a* mRNA were active in the preceding 25–40 minutes (event 1+ neurons) before euthanasia. Neurons expressing both *Arc* and *H1a* mRNA were active during both events (event 1+ and 2+ neurons) and are part of an overlapping neuronal ensemble.^42^^,^^43^ Independent cohorts of young adult and aged animals were counterbalanced and used for catFISH experiments. All catFISH experiments were conducted in the light phase of the day (8 a.m.–12 p.m.). Home cage animals (HC), left undisturbed in their home cages, were used to determine basal levels of mRNA expression. All other animals were subjected to events 1 (for 3 minutes) and 2 (for 2 minutes), separated by a 30-minute interval. As previously described, event 1 consisted of weak CFC training in context A. Following training, young adult animals were randomly assigned to distinct event 2 groups: Control (standard cage), Reactivation (Ctx A), new encoding (New Ctx B) or new encoding in the novel room (New Ctx B). Aged animals were subjected to event 1 and then assigned to Control and New encoding groups. Immediately after event 2, animals were transported to the euthanasia room (approximately 1 additional minute) and euthanized for *in situ* hybridization procedures (*SI Appendix*, Figure S6). The groups for catFISH experiments were chosen based on our behavioral data. The same experimenters conducted the studies in a similar environment, using similar procedures and animals with similar genetic backgrounds. Consistency in tissue preparation, catFISH procedures, and cellular imaging acquisition and quantification was monitored and verified across cohorts in the lab.

#### RNAscope *in situ* hybridization

After completing behavioral experiments designed to evaluate mRNA expression, animals were decapitated, and their brains were rapidly extracted, embedded in a cryoprotectant solution (OCT - Tissue-Tek) and stored at −80°C. Subsequently, coronal sections (20 μm thick) of the dorsal and ventral hippocampus were collected on adhesive slides (Surgipath X-tra, Cat#3800202, Leica Biosystems Richmond, Inc.) using a cryostat (Leica CM1850, Leica Biosystems Richmond, Inc.). The RNAscope Fluorescent Multiplex Assay (Advanced Cell Diagnostics, Cat#323133) was conducted following the manufacturing instructions for fresh-frozen tissue hybridization. Briefly, sections were fixed in 4% PFA for 15 minutes, dehydrated in ascending concentrations of ethanol (50%, 70%, and 100%) for 5 minutes each at room temperature, and air-dried. Subsequently, sections were incubated with protease solution (protease IV) at room temperature for 30 minutes and then washed with 1× PBS. After washing, target probes for *Arc* (GenBank accession number NM_019361.1; target region, 1519–2621, full-length *Arc*) and *H1a* mRNA (target region 68–719, 3′-UTR-) were applied (40 μL to each section) to the slides at 40°C for 90 minutes in a water bath for hybridization. After washing off slides in 1× Wash Buffer, the slides were incubated with preamplifier and amplifier probes (AMP1, 40°C for 30 minutes; AMP2, 40°C for 15 minutes; AMP3, 40°C for 30 minutes). The slides were then incubated with fluorescently labeled probes by selecting a specific combination of colors associated with each channel: green (Alexa 488 nm) and red (Alexa 550 nm) (AMP4 Alt4, 40°C for 15 minutes) to detect *Arc* and *H1a* in red and green, respectively. Finally, sections were incubated for 30 seconds with DAPI. After air-drying, slides were covered with a fluorescent mounting medium (Fluoromount TM Aqueous Mounting Medium; #F4680, Sigma-Aldrich) and were stored in the dark at 4°C until imaging.

#### Image acquisition and cell counting

Images were acquired using a confocal microscope (Leica SP8 AOBS Tandem Scanner) with a 40× oil objective. The photomultiplier tube (PMT) gain, pinhole sizes, and contrast settings were kept constant across all images. Confocal z-stacks (1 μm thickness) were acquired from two sections obtained from each animal (n = 3–4, per group), with two imaging fields in the dCA1 and vCA1 regions. To avoid stereological accounts, sections were collected with a 60 μm interval around bregma −3.8 mm for dCA1 and -5.8 mm for vCA1. The quantification of neuronal nuclei (DAPI+), nuclear *H1a* foci (*H1a*+), and nuclear *Arc* foci (*Arc*+) was performed manually using ImageJ/Fiji software ((https://imagej.nih.gov/ij/), NIH, Bethesda, MD). The mean quantified neurons across regions and experimental conditions are presented as supplementary data (*SI Appendix*, Tables S3 and S4). Neurons were classified as negative (no nuclear transcription foci), *H1a*+ (containing only *H1a* foci), *Arc*+ (containing only *Arc* foci) or *H1a*+/*Arc*+ (containing both *H1a* and *Arc* foci). Percentage of *H1a*+ neurons (total *H1a*+ neurons/total DAPI+ neurons), *Arc*+ neurons (total *Arc*+ neurons/total DAPI neurons) and *H1a*+/*Arc*+ neurons (*H1a*+/*Arc*+ neurons)/total DAPI+ neurons) were calculated. The percentage of chance overlap was calculated as (*H1a*+ neurons/DAPI+ neurons) x (*Arc*+ neurons/DAPI+ neurons). All cell counting was performed by a researcher blinded to the experimental groups to ensure unbiased results.

#### Behavioral analysis

A camera was positioned in front of the chambers and was connected to a computer. Behavior was recorded with ANY-maze software (Version 7.1, Stoelting Co., USA). Researchers were blind to the experimental groups during offline analysis. The percentage of time spent freezing was used as an index of fear memory. Freezing behavior was defined as body immobility and absence of vibrissae movements lasting 2 s or longer.

### Quantification and statistical analysis

#### Statistical analysis

The data were submitted to normality (Kolmogorov-Smirnov) and homogeneity of variance (Bartlett and Brown– Forsythe) tests. For datasets meeting these assumptions, Student’s t-tests (one or two-tailed), and analysis of variance (one or two-way ANOVA) followed by Bonferroni’s multiple comparisons test were employed. For datasets violating these assumptions, Wilcoxon or Mann-Whitney (two-tailed) tests and Kruskal–Wallis with Dunn’s post-hoc tests were used. Data were analyzed using GraphPad Prism version 8.0 (GraphPad Software, San Diego, USA), with significance levels set at *p* < 0.05. Data are presented as means ± standard error of the mean (SEM). No statistical methods were used to determine sample sizes, but our sample sizes were based on previous studies.^49^
