# Supplementary material for: Linking new information to a short-lasting memory trace induces consolidation in the hippocampus
Source: iScience. 2024 Nov 5;27(12):111320. doi: 10.1016/j.isci.2024.111320 (PMC11617307; doi:10.1016/j.isci.2024.111320)
Supplement: Document S1. Figures S1–S10, Tables S1, and S2 [file mmc1.pdf]

iScience, Volume ■■

Supplemental information

Linking new information to a short-lasting memory trace induces consolidation in the hippocampus

Andressa Gabriela Soliani, Jessica Santos Baptista, Beatriz Gangale Muratori, Lucia Armelin Correa, and Suzete Maria Cerutti

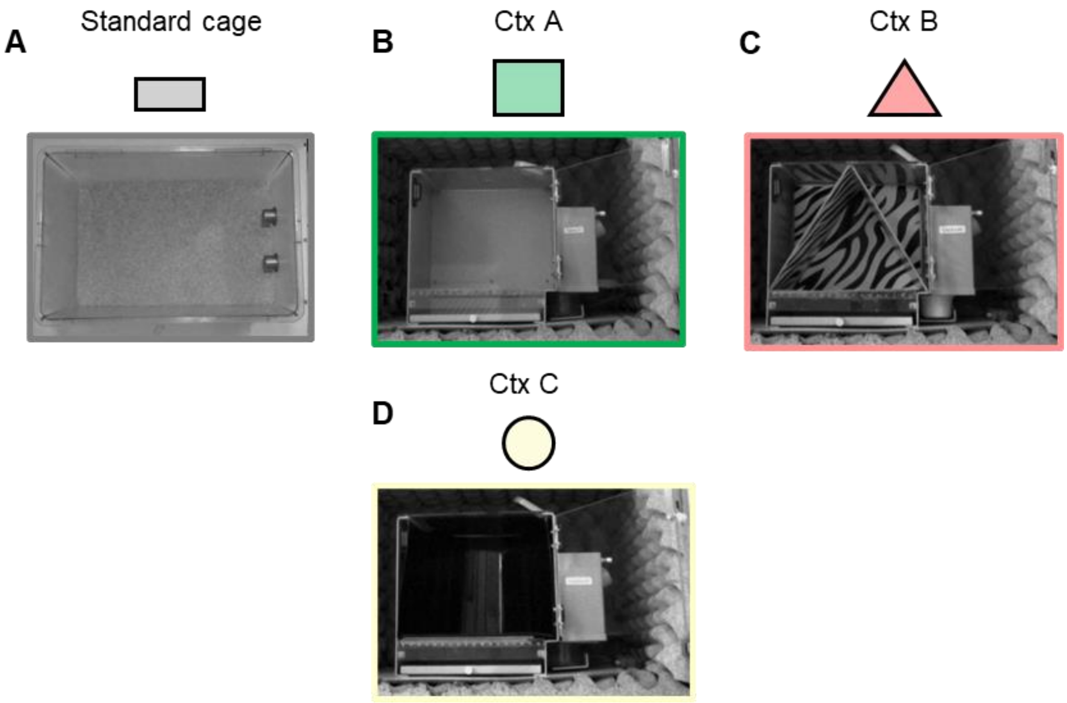

Fig. S1. Design of the distinct contexts used in the study. (A) Standard cage. (B) Context A. (C) Context B. (D) Context C.

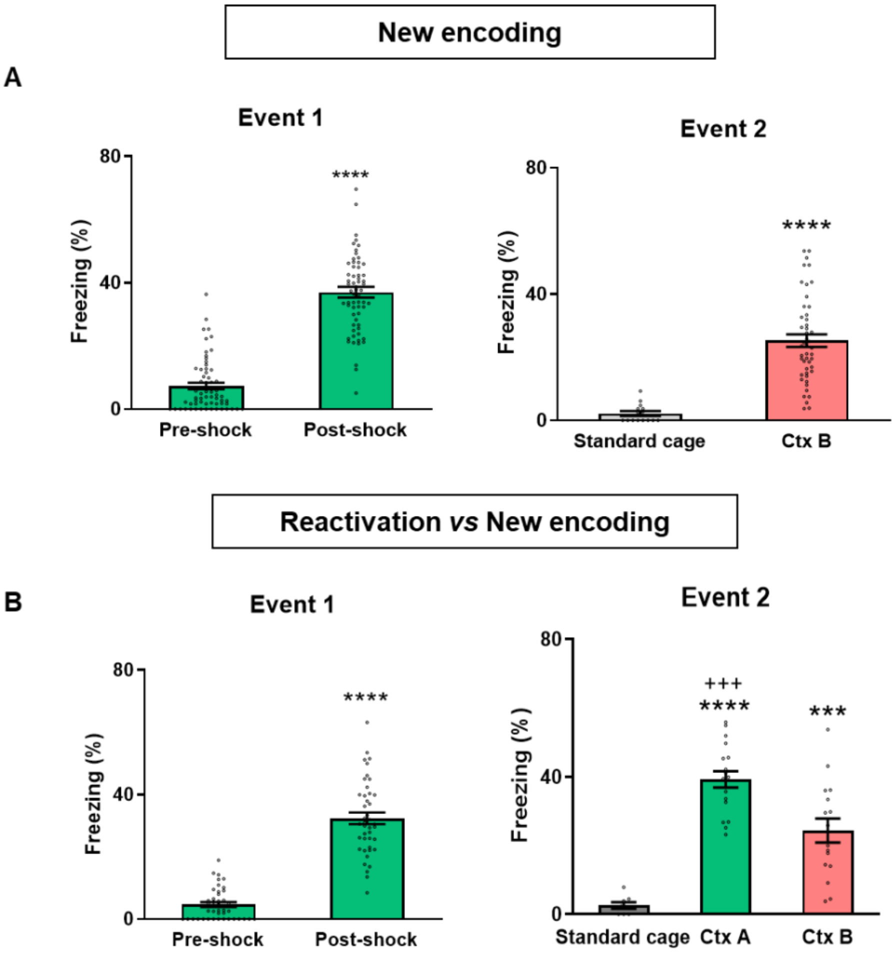

Fig. S2. Freezing levels during events 1 and 2. All animals underwent weak CFC training (Event 1) and, 30 min later, were assigned to distinct behavioral groups (Event 2). (A) Freezing levels during Event 1 (n=63). ****P<0,0001, according to two-tailed Wilcoxon test. Freezing levels during Event 2 (standard cage or Ctx B, n=15-48 per group). ****P<0,0001, according to the Mann-Whitney test. (B) Freezing levels during Event 1 (n=40), ****P<0,0001, according to two-tailed Wilcoxon test. Freezing levels during Event 2 (standard cage, Ctx A or Ctx B, n=8-16 per group). ****P<0,0001, *** P<0,001, compared to the standard cage group, +++ P<0,001, compared to the context B group, according to one-way ANOVA followed by Bonferroni's multiple comparisons test. Non-significant values are not shown. Data are presented as mean± S.E.M. and individual data plots.

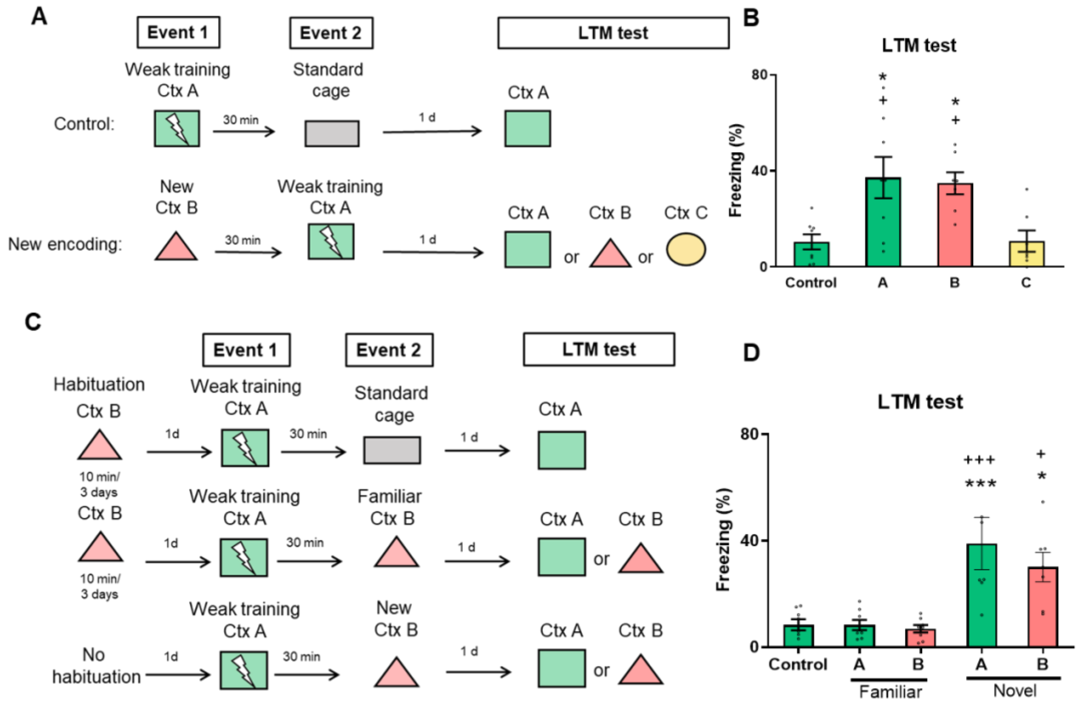

Fig. S3. Freezing levels in the LTM test. *Top panel* (A) Schematic representation for the experiments depicted at (B). Animals were given event 2 before event 1, separated by a 30-minute interval. Event 2 consisted of exposure to the either new context B or the standard cage. Event 1 consisted of a weak training protocol. On the next day, animals were tested for LTM in contexts A, B or C (n=7-8 per group). (B) Freezing levels for each group in the LTM test. *P < 0.0 compared to the control, + P<0.05, compared to the group tested in context C, according to one-way ANOVA followed by Bonferroni's multiple comparisons test. *Bottom panel* (C) Schematic representation for the experiments depicted at (D). Animals were habituated to context B for 3 days (10 minutes/day). Then, they received event 1 and event 2 with a 30-minute interval. As event 2, animals were exposed to the familiar context B, to the new context B (not habituated), or the standard cage. On the next day, they were tested for LTM in contexts A or B (n=7-8 per group). (D) Freezing levels for each group in the LTM test. *** P < 0.001, * P < 0.05 compared to the control, according to one-way ANOVA followed by Bonferroni's multiple comparisons test. +++P < 0.001, + P < 0.05, compared to animals that explored the familiar context B, according to two-way ANOVA followed by Bonferroni's multiple comparisons test. Non-significant values are not shown. Data are presented as mean± S.E.M. and individual data plots.

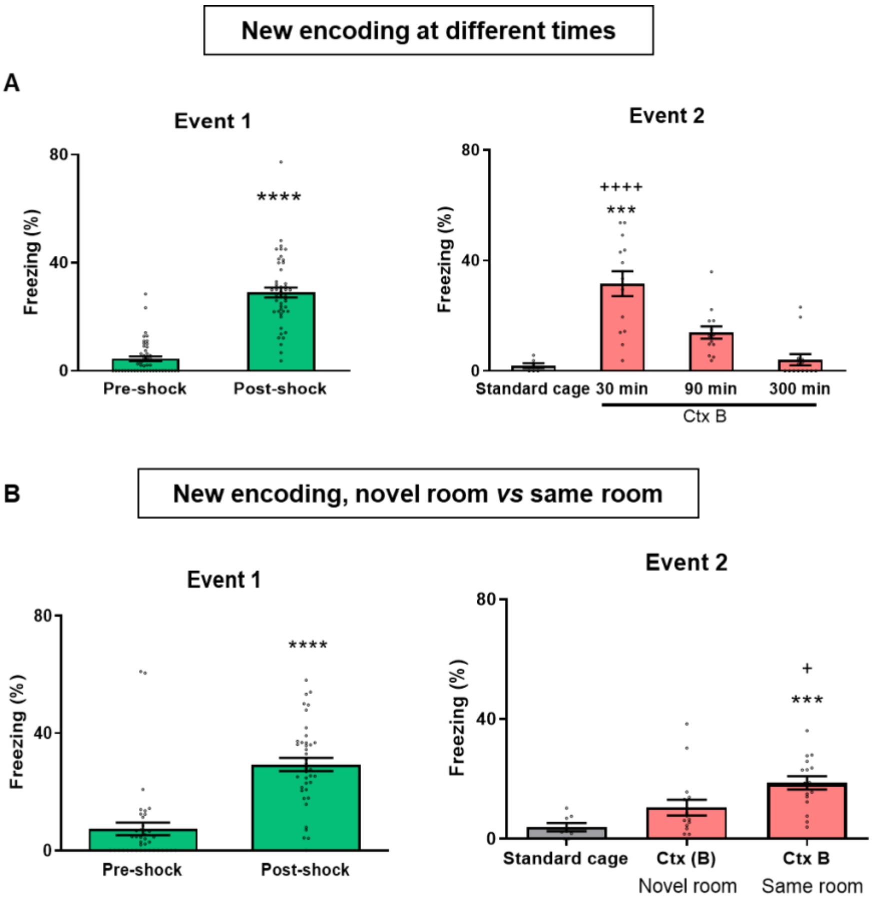

Fig. S4. Freezing levels during events 1 and 2. All animals underwent weak CFC training (Event 1) and were then assigned to distinct behavioral groups (Event 2). (A) Freezing levels during Event 1 (n=49). ****P<0,0001, according to two-tailed Wilcoxon test. Freezing levels during Event 2 (standard cage or Ctx B at 30, 90, or 300 min after event 1, n=7-14 per group). *** P<0,001, compared to the standard cage group, ++++ P<0,001, compared to the group exposed to Ctx B 300 min after training, according to the Kruskal-Wallis test followed by Dunns multiple comparisons test. B) Freezing levels during Event 1 (n=40), ****P<0,0001, according to two-tailed Wilcoxon test. Freezing levels during Event 2 (standard cage, ctx B within a novel room or within the same room, n=8-16 per group). *** P<0,001, compared to the standard cage group, + P<0,01, compared to the group exposed to context B in a novel room, according to the Kruskal-Wallis test followed by Dunn's multiple comparisons test. Non-significant values are not shown. Data are presented as mean± S.E.M. and individual data plots.

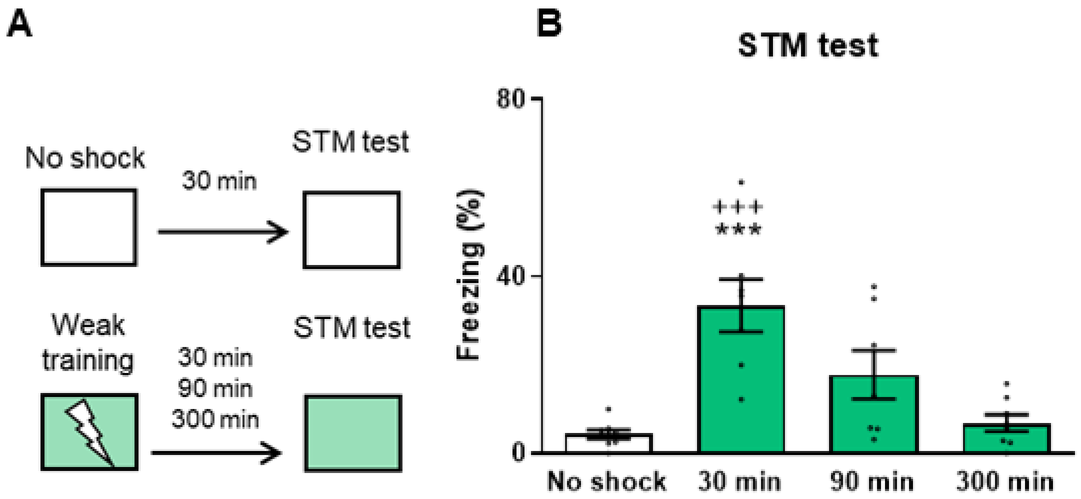

Fig. S5. **Freezing levels in the STM test**. (A) Schematic representation for the experiments depicted at (B). Animals were given weak training in context A and then tested for STM in different time intervals (30, 90 or 300 minutes after training) in the same context (n=7 per group). (B) Freezing levels in the STM tests. *** P < 0.001, compared to the no shock group, +++ P < 0.001, compared to the group tested at 300 minutes, according to one-way ANOVA followed by Bonferroni's multiple comparisons test. Non-significant values are not shown. Data are presented as mean± S.E.M. and individual data plots.

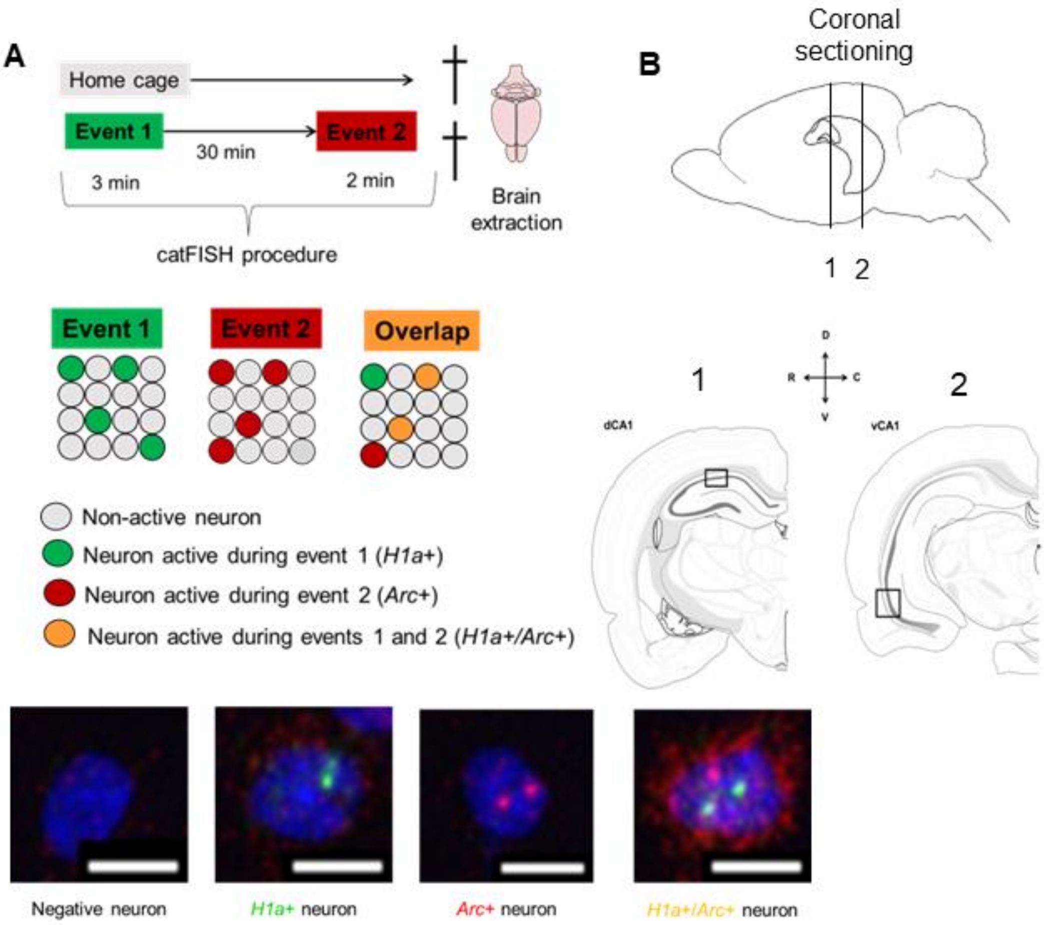

Fig. S6. Schematic representation of the cellular compartment analysis of temporal activity by fluorescent *in situ* hybridization (catFISH) experiments. *Left panel* (A) CatFISH procedure. Events 1 (3 min) and events 2 (2 min) were separated by a 30-min interval, whereas Home Cage animals remained undisturbed in the cage. Brains were extracted immediately after Event 2. Representation of a negative neuron (no *H1a* or *Arc* mRNA expression), neuron expressing intranuclear *H1a* (*H1a*+ neuron, in green), neuron expressing intranuclear *Arc* (*Arc*+ neuron, in red), and neuron expressing both *H1a* and *Arc* mRNA (*H1a+/Arc*+ neuron, in orange). Scale bar, 10 µm. *Right panel* (B) Schematic representation of the coronal sections of the hippocampus used for catFISH analysis. The squares in the illustration represent the location of microphotographs for dCA1 and vCA1.

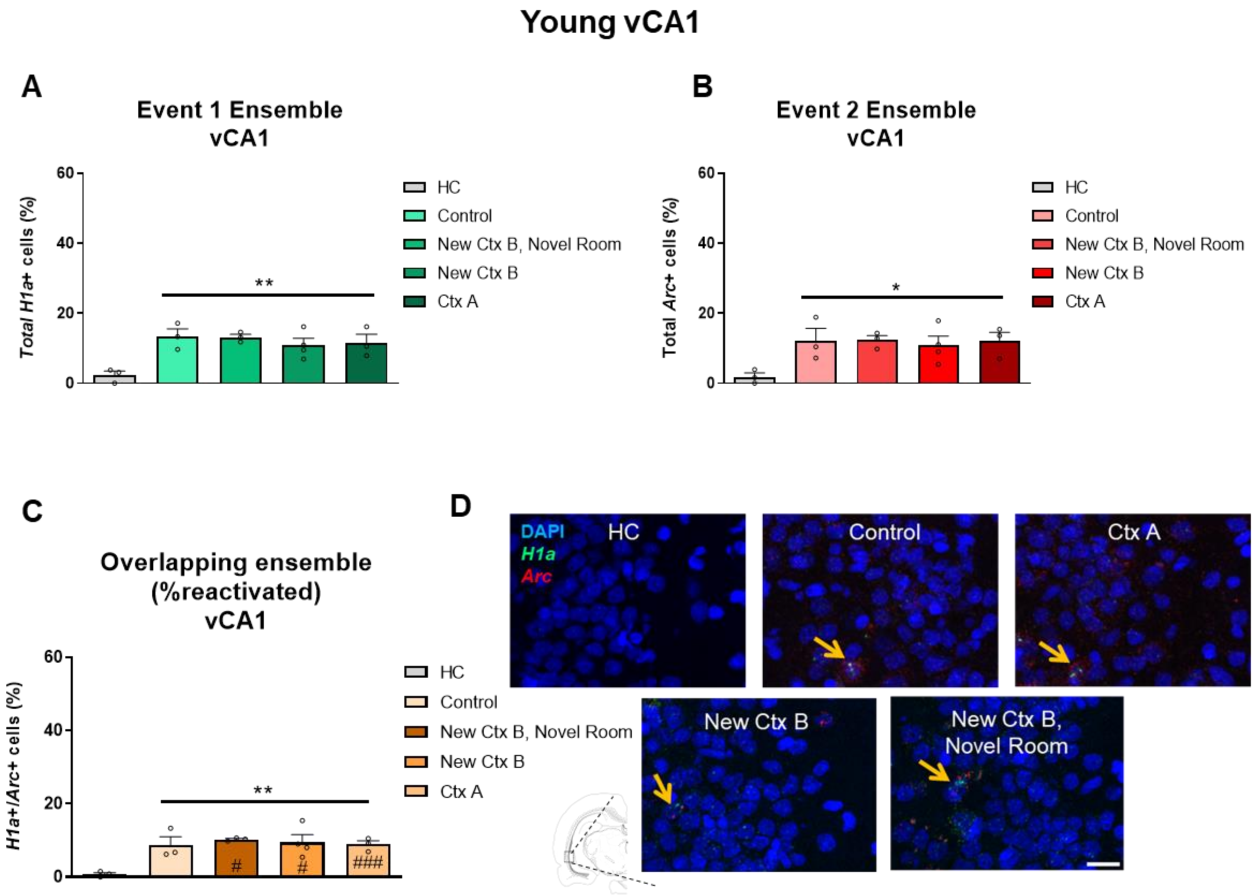

Fig. S7. Ensemble activity in the vCA1 region of the young hippocampus. A) Percentage of active neurons (total *H1a*+ neurons, ensemble 1) in vCA1. (B) Percentage of active neurons (total *Arc*+ neurons, ensemble 2) in vCA1 (C) Percentage of active neurons during events 1 and 2 (*H1a+*/*Arc*+ neurons, overlapping neuronal ensemble) in vCA1. (D) Representative microphotographs of neuronal ensembles in vCA1 for each group. n=3-4 animals per group, **** P < 0.0001, *** P < 0.001, ** P < 0.01, * P = 0.05, according to one-way ANOVA followed by Bonferroni's multiple comparisons test. # indicates a significant difference between the percentage of overlap and its chance level. ### P < 0.001, ## P < 0.01, # P = 0.05, according to one-sample t-test. *H1a*, *Arc* and DAPI staining are shown in green, red, and blue, respectively. Orange arrowheads indicate *H1a+/Arc*+ cells. Scale bar, 20 µm. Non-significant values are not shown. Data are presented as mean± S.E.M. and individual data plots.

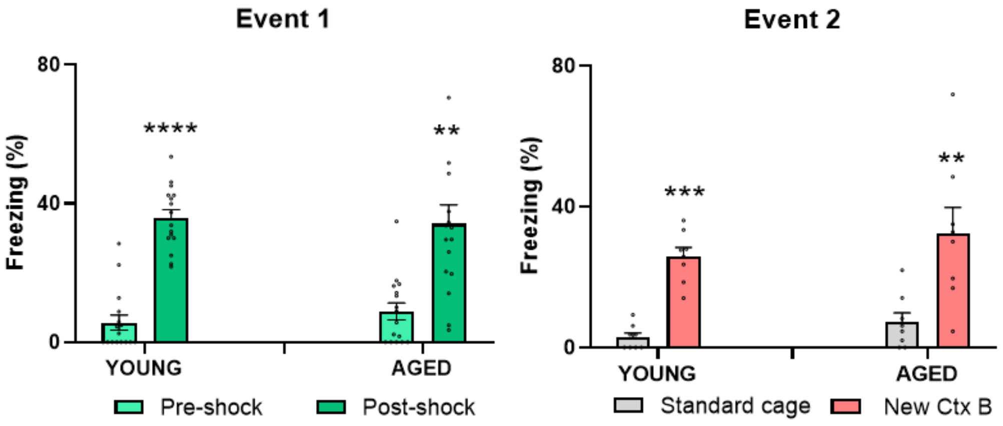

Fig. S8. Freezing levels during events 1 and 2 for young and aged animals. All animals underwent weak CFC training (Event 1) and, 30 min later, were assigned to distinct behavioral groups (Event 2). Freezing levels during Event 1, ****P<0,0001, **P<0,001 according to two-sided paired t-test (n=16 per group). Freezing levels during Event 2 (standard cage or context B, n=8 per group). *** P<0,001, according to Mann-Whitney test, ** P<0,001, according to two-sided unpaired t-test. Non-significant values are not shown. Data are presented as mean± S.E.M. and individual data plots.

Young vs Aged vCA1

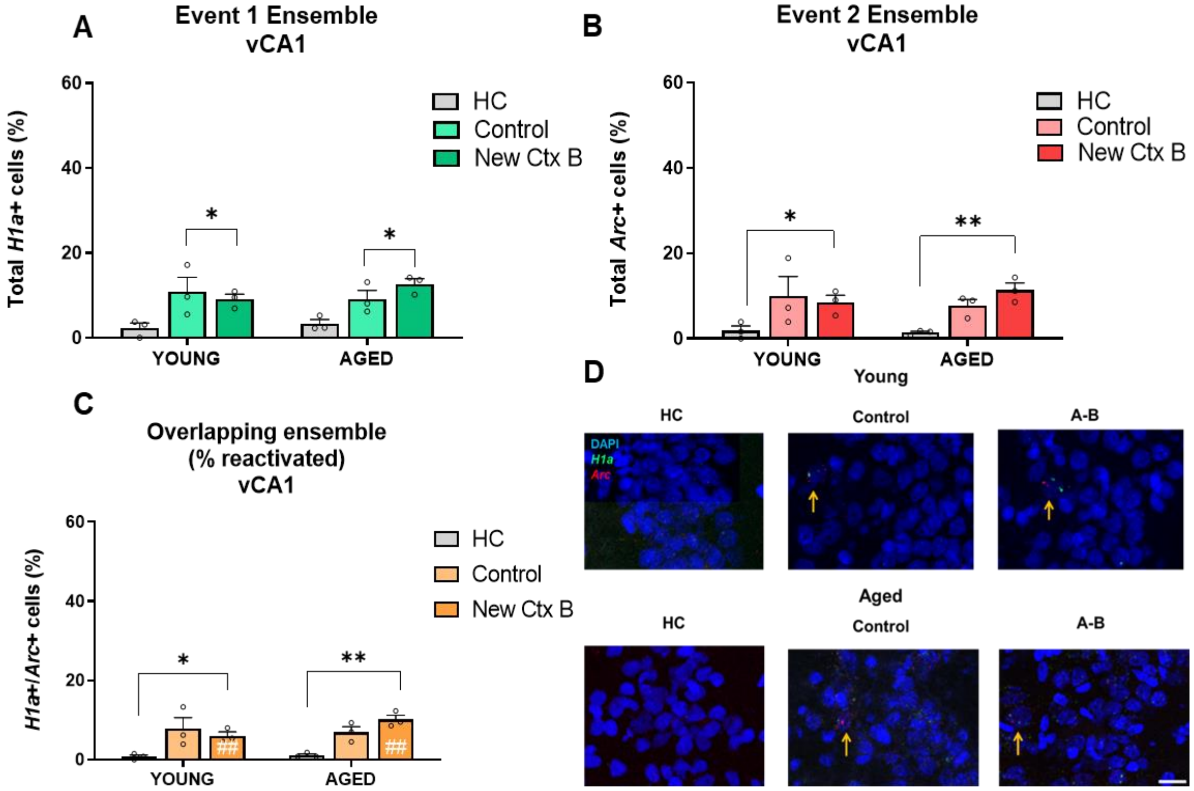

Fig. S9. Ensemble activity in the vCA1 region of both young and aged hippocampus. (A) Percentage of active neurons (total *H1a*+ neurons, ensemble 1) in vCA1. (B) Percentage of active neurons (total *Arc*+ neurons, ensemble 2) in vCA1 (C) Percentage of active neurons during events 1 and 2 (*H1a*+/*Arc*+ neurons, overlapping neuronal ensemble) in vCA1. (D) Representative microphotographs of neuronal ensembles in vCA1 for each group. *H1a*, *Arc* and DAPI staining are shown in green, red, and blue, respectively. Orange arrows indicate *H1a*+/*Arc*+ cells. Scale bar, 20 µm. ** $P < 0.01$, * $P < 0.05$, in comparison to the home cage group, according to one-way ANOVA followed by Bonferroni's multiple comparisons test. # indicates a significant difference between the percentage of overlap and its chance level. ## $P < 0.01$, according to one-sample t-test. Non-significant values are not shown. Data are presented as mean± S.E.M. and individual data plots.

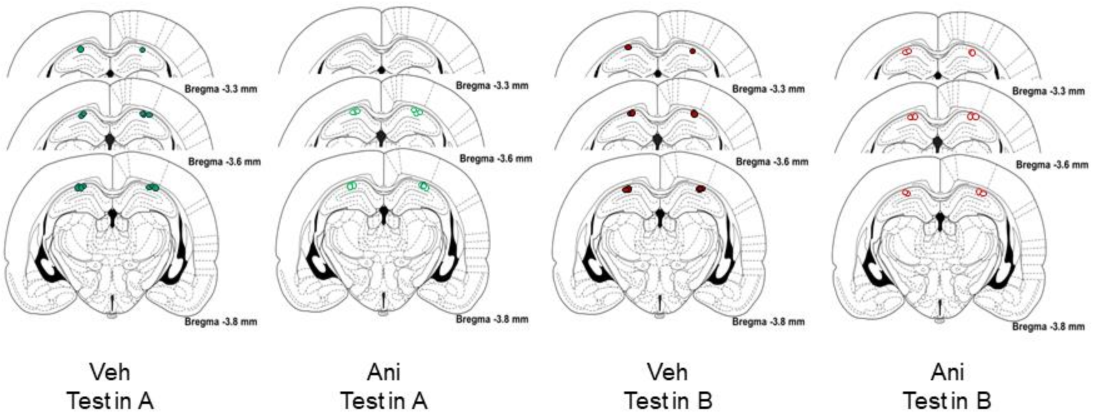

Fig. S10. Cannula tip placement in rats infused with vehicle (veh) or anisomycin (ani) following exposure to new context B. Coronal sections of the hippocampus showing cannula tip placements for individual animals in each group: test in context A, vehicle (veh, n = 8); test in context A (ani, n = 7); test in context B (veh, n = 8); test in context B (ani, n = 8).

Table S1. The table shows the mean percentage of freezing (±S.E.M) observed for each group during context exposure (without prior CFC training) or immediate shock experiments. n= number of animals. No difference among contexts was found (Kruskal-Wallis test, H=0.5345, P=0.7655), indicating that contexts used have no natural significance and are equivalent across animals. During immediate shock experiments, no significant difference among groups was found (one-way ANOVA, F (2, 18) = 3.286, P=0.0608).

Contexts		
**Groups**	**% time spent freezing Mean (±S.E.M)**	**N per group**
**Context A**	1,031±0,5313	8
**Context B**	2,307±1,182	8
**Context C**	1,805±0,9496	8
**Inmediate shock**		
**Groups**	**% time spent freezing Mean (±S.E.M)**	**N per group**
**Control**	4,765±1,887	7
**Test in A**	11,45±2,646	7
**Test in B**	5,649±1,218	7

Table S2. Mean number of neuronal nuclei (DAPI+ neurons), *H1a*+ only, and *Arc*+ only cells (±SEM) in the dCA1 and vCA1 regions of young adult and aged rats. n= number of animals. No significant differences were found among the young groups in cell counting for each region (dCA1: one-way ANOVA, F (4, 11) = 1.905, P=0.1798; vCA1: one-way ANOVA, F (4, 11) = 0.4962, P=0.7391). Similarly, no significant differences were found among the aged groups (dCA1: one-way ANOVA, F (2, 6) = 2.754, P=0.1417; vCA1: one-way ANOVA, F (2, 6) = 1.071, P=0.4002). On average, the number of neurons counted was similar in young and aged animals (dCA1: two-way ANOVA, age: F (1, 2) = 2.990, P=0.2259; group: F (2, 4) = 0.9514, P=0.4592; interaction age x group: F (2, 4) = 1.479, P=0.3306; vCA1: two-way ANOVA, age: F (1, 4) = 3.230, P=0.1467; group: F (1,039, 4,158) = 0.8441, P=0.4133; interaction age x group: F(2, 8) =1.826, P=0.2223).

**dCA1**				
**Group**	DAPI+	*H1a*+ only	*Arc*+ only	N per group
**Home cage**	100,1±2,454	1,5 ±0,8386	2,20 ± 1,400	3
**Control**	96,93±2,945	18,47± 2,195	15,33 ± 5,206	3
**Ctx A**	100,1±0,3180	6,73 ±0,8452	5,00 ± 1,206	3
**New Ctx B**	105,4 ± 4,035	10,38±2,819	12,90 ± 2,137	4
**New Ctx B in novel room**	94,00 ± 3,617	20,77±5,746	24,27 ± 4,964	3
**Aged Home cage**	98,6 ±1,358	3,40 ± 1,159	1,03 ± 0,606	3
**Aged Control**	92,97±2,088	13,66 ± 2,677	4,13 ± 1,683	3
**Aged New Ctx B**	100,3±3,121	8,70 ± 2,196	5,53 ± 1,530	3
**vCA1**				
**Group**	DAPI+	*H1a*+ only	*Arc*+ only	N per group
**Home cage**	124,6 ± 5,856	1,533±0,7667	1,267±0,8969	3
**Control**	128,8 ± 2,936	4,667 ±0,9735	2,575±1,276	3
**Ctx A**	127,1 ± 2,022	2,667±1,519	3,000±1,735	3
**New Ctx B**	129,6 ± 4,500	2,525±0,8664	2,525±0,9232	4
**New Ctx B in novel room**	123,0 ± 2,309	2,933±1,099	2,033±1,065	3
**Aged Home cage**	123,6 ± 3,789	1,967±0,3667	1,133±0,3333	3
**Aged Control**	115,0 ± 6,372	3,000±0,7024	4,800±1,721	3
**Aged New Ctx B**	113,5 ± 5,248	2,633±0,7333	8,733±1,837	3
